# Beyond Analyte Recovery: A Holistic Review of Conventional and Miniaturized Extraction Techniques for Pharmaceutical Analysis in Environmental Samples

**DOI:** 10.1021/acsomega.6c04664

**Published:** 2026-09-04

**Authors:** Masimba Tapera, Tshimangadzo S. Munonde, Gcina Mamba, Ramakwala C. Chokwe, Lawrence M. Madikizela

**Affiliations:** † Unisa Science Campus, Institute for Nanotechnology and Water Sustainability, University of South Africa, Private Bag X6, Johannesburg 1709, Gauteng, South Africa; ‡ Unisa Science Campus, Department of Chemistry, University of South Africa, Private Bag X6, Johannesburg 1709, Gauteng, South Africa

## Abstract

The presence of pharmaceuticals in the environment has become a significant analytical and ecological concern because of their continuous release, persistence, and potential adverse effects. Their occurrence at trace levels, combined with the complexity of environmental matrixes, necessitates efficient extraction and preconcentration prior to instrumental determination. This review critically evaluates the performance of conventional extraction techniques, namely, liquid–liquid extraction (LLE) and solid-phase extraction (SPE), compared with their miniaturized versions for the extraction and preconcentration of pharmaceuticals in environmental samples. Representative peer-reviewed studies were critically evaluated, focusing on reported extraction recoveries, enrichment factors, and practical advantages and limitations. Conventional LLE and SPE have recoveries ranging from 9 to 120% and are limited by higher solvent consumption and longer processing times. Miniaturized techniques generally achieved improved enrichment (7.3–273.0-fold enrichment) and recoveries between 9.4 and 229.0% while significantly reducing solvent usage and enhancing sensitivity. Recoveries exceeding the accepted range may reflect matrix effects or analytical bias and should, therefore, be interpreted with caution. Beyond recovery and enrichment trends, this review also synthesizes and compares extraction techniques based on practical performance characteristics, including simplicity, rapidness, selectivity, versatility, greenness, and reusability to support informed method selection. While miniaturized techniques excel in rapidness, greenness, and selectivity, the conventional techniques remain the best in terms of simplicity and versatility. The review confirms that no single extraction technique can be regarded as universally superior. Therefore, method selection should balance the analytical performance with operational practicality, environmental sustainability, and cost. Continued improvement of extraction techniques is recommended to further enhance the pharmaceutical monitoring in environmental systems.

## Graphical abstract

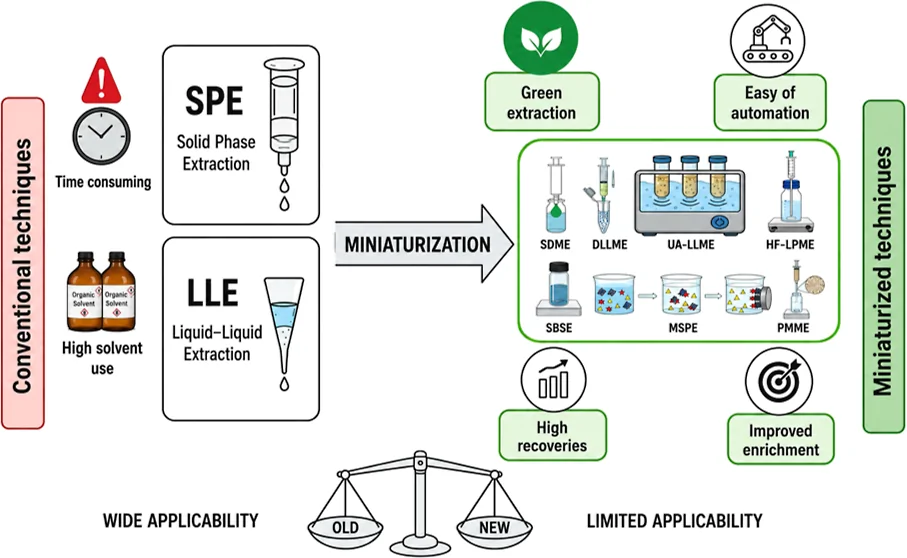

## 1 Introduction

The occurrence of pharmaceuticals in the environment dates back to the 1970s, when steroid hormones were first detected in river water.^1,2^ Over time, pharmaceuticals have become significant environmental pollutants. The increase in population and technological advancement, especially in the manufacturing sector, has promoted an increase in the volume of pharmaceuticals discharged into the environment.^3^ Pharmaceuticals are a unique group of environmental pollutants because of their physical and chemical properties.^4^ More than 100 pharmaceutical compounds have been detected in sewage influent, sewage effluent, surface water, groundwater, food crops, and aquatic organisms.^5,6^

The root sources of pharmaceuticals in the environment are sewage disposal systems^7^ as well as improper disposal of drugs and landfill leachate.^8,9^ Despite being ubiquitous environmental pollutants, pharmaceutical concentrations in the environment are usually in the ng/L to μg/L range, relatively lower than other contaminants such as potentially toxic metals and microbes.^10^ Due to their occurrence at trace concentration levels, the accurate detection and quantification of pharmaceuticals in environmental samples is usually challenging.^11^ Additionally, the complex nature of environmental matrices makes analytical detection and quantification difficult, with the need for an initial thorough sample preparation and preconcentration step(s).^12^ Sample preparation serves to make environmental samples compatible with analytical instruments by removing interfering matrix components and enriching the target analytes/pharmaceuticals to detectable levels.^13,14^

Traditional sample preparation has relied on liquid–liquid extraction (LLE) and solid-phase extraction (SPE) techniques.^14^ However, these conventional methods have significant drawbacks. LLE uses large volumes of organic solvents that are often toxic and hazardous to both the environment and operators.^15^ While SPE uses smaller solvent volumes than LLE, it remains laborious and time-consuming and often lacks selectivity, leading to co-extraction of interfering substances such as humic materials.^16^ The limitations of these conventional techniques have driven the development of microextraction methods that achieve high enrichment factors while minimizing solvent consumption and reducing waste.^15^ This evolution was originally motivated by the reduction or elimination of organic solvents and was initiated by the invention of solid-phase microextraction (SPME) and liquid-phase microextraction (LPME).^17^ Since then, various miniaturized microextraction techniques have been developed to improve performance during sample preparation, particularly in complex environmental water samples such as wastewater, surface, ground, sea, and river waters.^18^

Over the past three decades, pharmaceutical extraction techniques have evolved from conventional solvent-intensive methods to more efficient and miniaturized approaches aimed at reducing solvent consumption, improving automation, and enhancing the analytical performance. Although several review articles have examined this transition, they differ in scope and depth, leaving important gaps in the comparative evaluation of the extraction performance. For instance, Daniels et al.^19^ provided a comprehensive overview and transition of conventional, miniaturized, and online extraction techniques for environmental water samples, but their analysis was largely limited to specific pharmaceuticals such as carbamazepine and diclofenac, which restricts general applicability. Similarly, Lv et al.^20^ compared conventional and miniaturized sample preparation approaches, but the discussion was largely centered on non-steroidal anti-inflammatory drugs (NSAIDs) across multiple matrices, including biological fluids, environmental water, and food. The focus on NSAIDs narrows the broader relevance of their work. Ullah and Tuzen^21^ reviewed recent advancements in LLE and SPE miniaturization with broader application to organic and inorganic analytes, particularly metals, in drinking water, biological, environmental, and food samples. However, this broader focus of their review reduces detailed insight into the pharmaceutical-specific extraction performance. Camara et al.,^22^ on the other hand, emphasize methodological advancements and analytical performance, though their discussion remains largely technique-oriented with limited comparison of practical factors such as ease of use and rapidness.

In contrast, the present review specifically examines pharmaceutical extraction from environmental matrices and critically evaluates extraction performance based on reported recoveries and enrichment factors. The literature considered in this review was drawn from peer-reviewed studies published over the last two decades and retrieved from major scientific databases, including Scopus, Web of Science, ScienceDirect, PubMed, and Google Scholar. The literature search was guided by combinations of keywords such as pharmaceuticals, environmental samples, wastewater, surface water, extraction techniques, preconcentration, liquid–liquid extraction (LLE), solid-phase extraction (SPE), microextraction, dispersive liquid–liquid microextraction (DLLME), solid-phase microextraction (SPME), and hollow fiber liquid-phase microextraction (HF-LPME). Priority was given to studies providing clear methodological descriptions, recovery and/or enrichment factor data, and evidence that validated analytical methods with appropriate quality control procedures had been employed, while studies lacking sufficient analytical detail were excluded. As the primary objective of this review was to compare and critically assess extraction and preconcentration methods rather than to evaluate analytical method validation, detailed comparisons of validation parameters such as precision, accuracy, limits of detection, limits of quantification, and linearity were considered beyond the scope of this review. Consequently, the extraction efficiencies discussed herein are interpreted within the context of studies that have already established the analytical reliability of their respective methods.

To facilitate comparison across extraction techniques, a qualitative performance assessment was developed based on practical features consistently reported in the reviewed literature. The performance descriptors (e.g., simple, fast, broad applicability, environmentally friendly, reusable, cheap) reported by the original authors were synthesized and translated into qualitative categories (low, moderate, and high) according to predefined criteria (Table 1). These categories were subsequently assigned numerical scores of 1–3 solely for graphical visualization and comparison, where 1 = low, 2 = moderate, and 3 = high (Figure 8). The qualitative scores were assigned solely to facilitate visual comparison of the practical characteristics of the reviewed extraction techniques.

**Table 1:** Typical Practical Features of Selected Pharmaceutical Extraction Methods as Reported in Previous Studies^a^

Extraction method	Simplicity	Rapidness	Selectivity	Versatility	Greenness	Reusability	Cost	References
LLE	moderate	low	low	high	low	low	low	^59,60^
SDME	high	high	moderate	low	high	low	moderate	^61,62^
HF-LPME	moderate	moderate	high	moderate	high	low	moderate	^63,64^
DLLME	moderate	highs	moderate	moderate	moderate	low	moderate	^65,66^
UA-LLME	moderate	high	moderate	moderate	moderate	low	high	^67,68^
SPE	moderate	moderate	high (sorbent-dependent)	high	moderate	moderate	high	^69,70^
SPME	moderate	moderate	high	moderate	high	high	moderate	^71,72^
MSPE	moderate	high	high	moderate	moderate	moderate	high	^73,74^
SBSE	moderate	moderate	high	moderate	high	high	high	^75,76^
MSPDE	moderate	moderate	moderate	moderate	moderate	moderate	moderate	^77,78^
PMME	moderate	moderate	high	moderate	moderate	moderate	high	^79,80^

a Abbreviations: liquid–liquid extraction (LLE), single-drop microextraction (SDME), hollow fiber-liquid phase microextraction (HF-LPME), dispersive liquid–liquid microextraction (DLLME), ultrasound-assisted liquid–liquid microextraction (UA-LLME), solid-phase extraction (SPE), solid-phase microextraction (SPME), magnetic solid-phase extraction (MSPE), stir-bar sorptive extraction (SBSE), matrix solid-phase dispersion extraction (MSPDE), and polymer monolith microextraction (PMME). Interpretation: Simplicity: low = complex setup and multiple operational steps, moderate = moderate handling requirements, and high = simple setup with minimal handling. Rapidness: low = time-consuming extraction and processing, moderate = moderate extraction time, and high = rapid extraction and sample processing. Selectivity: low = limited performance with greater susceptibility to matrix interferences, moderate = moderate cleanup performance, high = strong analyte discrimination and efficient matrix cleanup. Versatility: low = limited analyte or matrix applicability, moderate = applicable to several analytes or matrices, and high = broad compatibility across pharmaceutical classes and environmental matrices. Greenness: low = high solvent consumption and/or use of hazardous solvents, moderate = reduced solvent use with partial improvement in environmental impact, high = minimal solvent consumption and use of greener extraction approaches. Reusability: low = single-use materials, moderate = limited reuse with some performance loss, high = multiple reuse cycles without major loss of extraction performance. Cost: low = relatively inexpensive consumables and operation, moderate = moderate operational cost, and high = comparatively expensive materials, instrumentation, or maintenance requirements.

This review narrows its scope to pharmaceuticals in environmental samples and directly compares conventional and miniaturized approaches using quantitative indicators of extraction efficiency and practicality. The main contribution of this review, therefore, lies in its targeted evaluation of recovery and enrichment trends and its emphasis on practical and sustainability-related criteria, including simplicity, rapidness, selectivity, versatility, greenness, and reusability. By integrating both performance metrics (recoveries and enrichment factors) and practical considerations, this review provides a more holistic evaluation of conventional and miniaturized extraction techniques. This broader assessment framework allows researchers to move beyond efficiency alone and consider operational feasibility and sustainability when selecting the appropriate methods for pharmaceutical monitoring in environmental systems. Overall, this critical narrative review synthesizes the representative literature to compare the analytical performance and practical applicability of the conventional and miniaturized extraction techniques.

## 2 Extraction and Preconcentration Performance Criteria

Environmental samples such as water, soil, and sediments typically contain analytes of interest at trace (parts per billion (ppb)) or ultratrace (parts per trillion (ppt)) levels.^23^ Direct analysis of these samples is often impossible because the concentrations fall below the detection capabilities of most analytical instruments.^24^ Additionally, environmental matrices are complex and contain numerous interfering compounds that can mask target analytes or cause signal suppression.^25^ Thus, extraction techniques are employed to isolate and preconcentrate the analytes of interest to detectable levels. In practice, extraction and preconcentration are performed by transferring analytes from a bulk sample matrix (aqueous or solid) into a smaller volume of solvent or sorbent phase through mechanisms such as partitioning, adsorption, or diffusion.^26^ The efficiency of this transfer is influenced by several critical factors, including analyte physicochemical properties (polarity, pKa, and hydrophobicity), matrix composition, pH, ionic strength, extraction time, temperature, solvent type, sorbent chemistry, and phase ratio.^16^ Poor optimization of these variables may negatively affect recovery, while careful control can significantly enhance the extraction efficiency and enrichment.

Recovery is the fundamental measure of extraction efficiency, representing the percentage of the target analyte successfully extracted from the original sample into the final extract.^16,27^ Recovery is calculated as the ratio of the amount of analyte recovered in the extract (*A*_ex_) to the initial amount present in the sample (*A*_o_), expressed as a percentage (eq 1), often expressed as a percentage.^28,29^

1 $$recovery\;\left(\%\right)=\frac{A_{ex}}{Ao}\times 100$$

where *A*_o_ is the total amount of the analyte in the sample and *A*_ex_ is the total amount of the analyte that is extracted into the extracted phase.

Acceptable recovery values typically range from 70 to 120% according to regulatory guidelines, with most successful methods achieving recoveries between 80 and 105%.^30,31^ It should be noted that recoveries exceeding 100% do not necessarily indicate a superior extraction efficiency. Such values may arise from matrix-induced signal enhancement, calibration bias, incomplete correction for matrix effects, or other analytical uncertainties and therefore often represent apparent rather than true extraction recoveries.^32^ Since this review compiles recovery values reported in the original peer-reviewed studies, the reported recoveries are presented as published by the respective authors. Consequently, recoveries above 100% should be interpreted cautiously and considered alongside other method performance characteristics, including precision, reproducibility, selectivity, and validation data, rather than as direct evidence of superior extraction performance.

Recovery is strongly influenced by extraction selectivity, solvent–analyte affinity, matrix interferences, and equilibrium conditions. For example, inadequate pH adjustment or insufficient extraction time can reduce analyte partitioning, while excessive co-extraction of matrix components may increase or suppress measured recoveries.^25^

The enrichment factor (EF) quantifies the concentration enhancement achieved through the extraction process. EF can be defined as the ratio of analyte concentration in the final extract to the initial concentration in the sample, expressed using eq 2.^28,33^

2 $$Enrichment\;factor=\frac{C_{fin}}{C_{ini}}$$

where *C*_fin_ is the analyte concentration in the final extract and *C*_ini_ is the initial concentration in the sample. Alternatively, EF can be calculated as the ratio of slopes of calibration curves obtained with and without preconcentration.^30,34^ High enrichment factors are crucial for improving analytical sensitivity, with successful methods typically achieving EF values ranging from 25 to over 400.^30,35^ However, EF is directly dependent on both recovery and the final extract volume; incomplete extraction or analyte loss during cleanup will lower EF, even when significant volume reduction is achieved. Techniques that combine strong selectivity with small elution volumes typically produce higher enrichment.^36^

Some researchers use the preconcentration factor (PF) to get an overview of the extraction performance. The PF represents the theoretical concentration increase based purely on volume reduction and is calculated as the ratio of initial sample volume to final extract volume using eq 3.^29,33,37^

3 $$Preconcentration\;factor=\frac{V_{s}}{V_{e}}$$

where *V*_s_ = sample volume and *V*_e_ = eluent volume. This parameter is independent of extraction efficiency and provides the maximum possible concentration enhancement if 100% recovery was achieved.^16^ EF values are usually lower than PF values because extraction is rarely exhaustive. Therefore, PF reflects the theoretical capacity, while EF reflects the actual performance, and recovery explains the gap between the two. These three parameters are inherently connected and are usually interpreted collectively, rather than in isolation. This review focuses primarily on extraction efficiency (recovery) and enrichment factors since they are directly linked to extraction performance. Among these, recovery receives greater emphasis because it is the most consistently reported performance metric in the reviewed literature and remains the principal indicator of extraction efficiency in analytical chemistry. Unless otherwise stated, the recovery, enrichment factors, and limits of detection (LODs) presented throughout this review were extracted from representative peer-reviewed studies and are reported as published by the original authors. The observed variability reflects differences in target pharmaceuticals, environmental matrices, extraction conditions, and analytical methodologies and should therefore be interpreted as representative performance rather than directly comparable values obtained under standardized experimental conditions.

Beyond these quantitative metrics, practical and sustainability-related considerations increasingly shape the selection of the extraction methods. A technique may provide high EF and acceptable recovery but score low in greenness due to high solvent consumption or use of toxic solvents or low in simplicity due to complex operational steps. Similarly, rapidness and reusability influence the laboratory throughput and long-term costs. Therefore, a balanced evaluation of analytical efficiency (recovery, EF, PF) alongside operational practicality (simplicity, selectivity, versatility) and environmental sustainability (greenness, reusability) provides a more holistic understanding of the extraction performance in environmental pharmaceutical analysis.

## 3 Conventional versus Miniaturized Pharmaceutical Extraction Techniques

Recent research shows that two main methods are used for pharmaceutical extraction: nearly 44% utilize conventional LLE and its miniaturized versions, while 56% use conventional SPE and its miniaturized versions.^38^ Conventional methods present significant limitations, which have driven the development of modern, miniaturized alternatives. LLE requires large volumes of organic solvents that are often toxic and hazardous to both the environment and operators.^13,15,22,39^ While SPE uses smaller solvent volumes than LLE, it remains laborious, time-consuming, and expensive.^40,41^ Additionally, traditional SPE sorbents often lack selectivity, resulting in the coextraction of interfering substances such as humic materials.^13^ The limitations of these conventional extraction techniques have prompted the development of alternative techniques. Among these new approaches, the miniaturized techniques have gained greater attention because of their advantages, including faster and more cost-effective extractions.

The common miniaturized versions of LLE include single-drop microextraction (SDME), hollow fiber-liquid phase microextraction (HF-LPME), and dispersive liquid–liquid microextraction (DLLME) (Figure 1). The miniaturized versions for SPE include SPME, magnetic solid-phase extraction (MSPE), matrix-solid-phase dispersion extraction (MSPDE), and stir-bar sorptive extraction (SBSE) (Figure 1). These miniaturized microextraction techniques offer numerous advantages over traditional methods, including speed, reliability, high sensitivity and selectivity, efficient sample cleanup, and significant enrichment factors, making them highly suitable for complex pharmaceutical analyses.^40,41^

**Figure 1:**
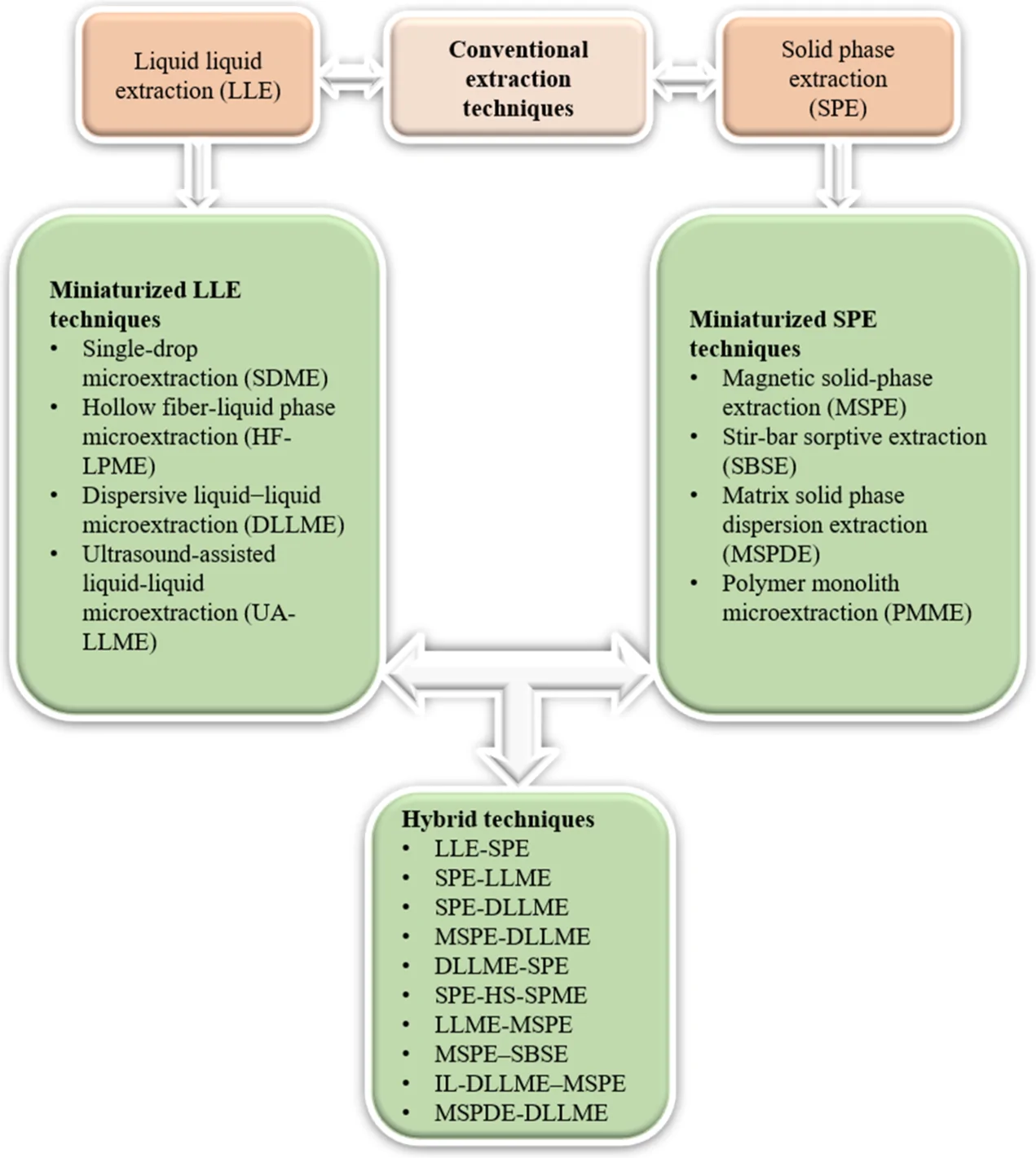
Common categories of pharmaceutical extraction techniques.

Moreover, these different extraction techniques can be modified or integrated to give hybrid approaches depending on the desired outcome (Figure 1). Hybrid approaches reported in literature include liquid–liquid extraction coupled with solid-phase extraction (LLE-SPE),^42^ solid-phase extraction coupled with liquid–liquid microextraction (SPE–LLME),^43^ solid-phase extraction coupled with dispersive liquid–liquid microextraction (SPE-DLLME),^44^ magnetic solid-phase extraction coupled with dispersive liquid–liquid microextraction (MSPE-DLLME),^45^ dispersive liquid–liquid microextraction coupled with solid-phase extraction (DLLME-SPE),^46^ solid-phase extraction coupled with headspace solid-phase microextraction (SPE–HS–SPME),^47^ liquid–liquid microextraction coupled with magnetic solid-phase extraction (LLME-MSPE),^48^ magnetic solid-phase extraction coupled with stir-bar sorptive extraction (MSPE-SBSE),^49^ ionic liquid-based dispersive liquid–liquid microextraction coupled with magnetic solid-phase extraction (IL-DLLME-MSPE),^50^ and matrix solid-phase dispersion coupled with dispersive liquid–liquid micro-extraction (MSPDE-DLLME).^51^

With continuous technological advancements, pharmaceutical extraction techniques have been evolving considerably. Sample preparation has progressed from conventional extraction methods to more modern, sustainable, and integrated analytical approaches that offer improved efficiency, selectivity, and environmental compatibility.^52^ Figure 2 summarizes the technological evolution of pharmaceutical extraction techniques, highlighting the progressive shift from conventional LLE^53^ and SPE^54^ to miniaturized,^55^ sorbent-based,^56^ green,^57^ and hybrid^58^ extraction techniques. Rather than representing isolated developments, the roadmap illustrates how advances in miniaturization, sorbent engineering, and extraction media have collectively improved the extraction efficiency while reducing solvent consumption and simplifying analytical workflows. More recent innovations emphasize the integration of green chemistry principles with hybrid extraction platforms and advanced materials, reflecting the growing demand for environmentally responsible, high-throughput, and field-deployable analytical methods. This evolutionary perspective provides the framework for understanding the comparative practical and sustainability characteristics discussed in subsequent sections.

**Figure 2:**
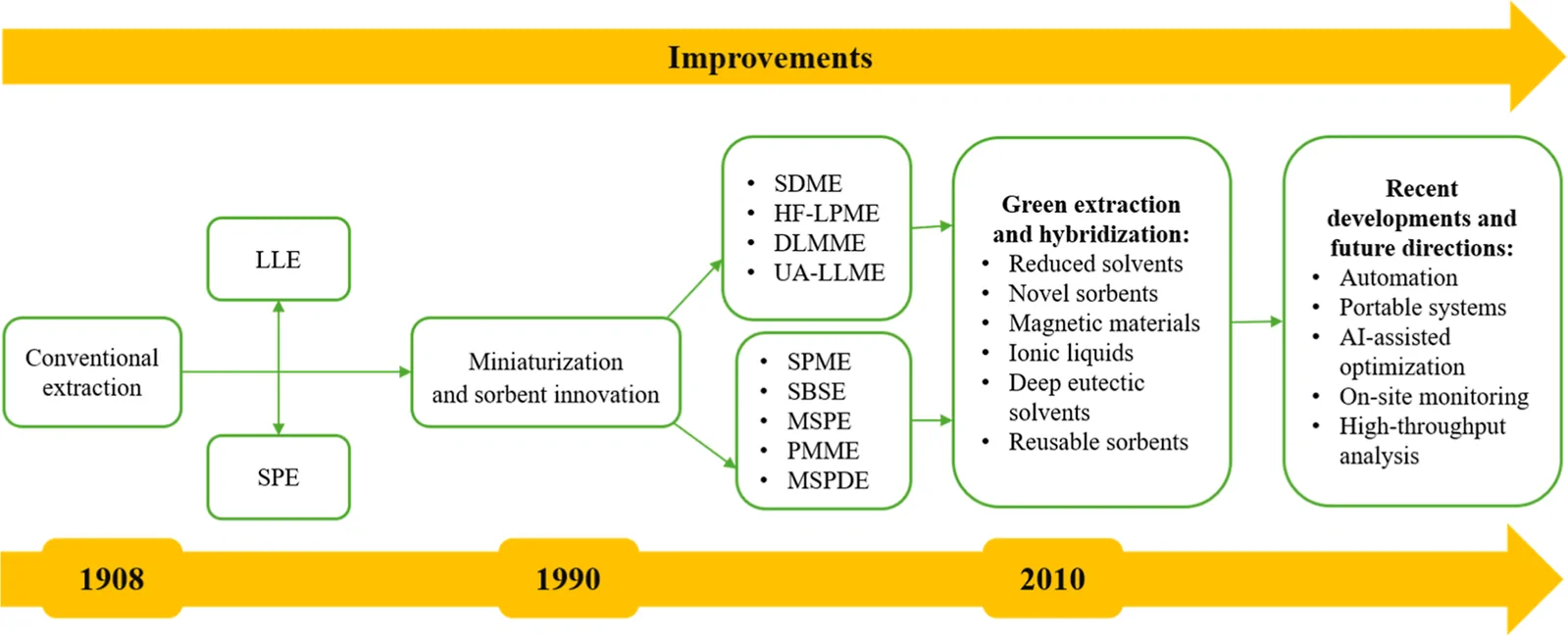
Representative evolutionary roadmap showing major technological milestones in pharmaceutical extraction for environmental analysis. Only selected techniques are presented to illustrate the general developmental trends, rather than to provide a comprehensive inventory of extraction methods.

Besides the basis of the principle of operation, extraction methods can be classified based on their practicality and environmental sustainability features. Some techniques can be described as simple, while others are complex, and some can be greener, while others are non-environmentally friendly. A summary of typical practical features of the conventional and miniaturized extraction techniques as reported in previous studies is given in Table 1. These practical features have been grouped into six classes: simplicity, rapidness, selectivity, versatility, greenness, reusability, and cost; where simplicity implies ease of setup and operation, rapidness means speed of extraction and processing, selectivity indicates the ability to friendliness, target analytes from the matrix, versatility indicates the range of analytes and sample types applicable, greenness shows the degree of solvent reduction and environmental friendliness, reusability indicates the ability to reuse sorbent/materials without a significant loss of performance, and cost gives a measure of monetary expenses needed for the materials, instrumentation, operation, and/or maintenance. The practical descriptors summarized in Table 1 were synthesized from representative studies cited for each extraction technique. The qualitative ratings (low, moderate, high) reflect the overall trends consistently reported in the literature rather than absolute performance values, results, or cost obtained under standardized experimental conditions. These descriptors formed the basis of the 1–3 scores presented graphically in Figure 8.

### 3.1 Liquid–Liquid Extraction and Miniaturized Versions

LLE and SPE have been commonly used as extraction methods of choice in pharmaceutical analysis.^81,82^ LLE is a method that entails the partitioning of a compound between two immiscible liquids or phases, each possessing distinct solubilities for the compound,^83,84^ depicted in Figure 3.

**Figure 3:**
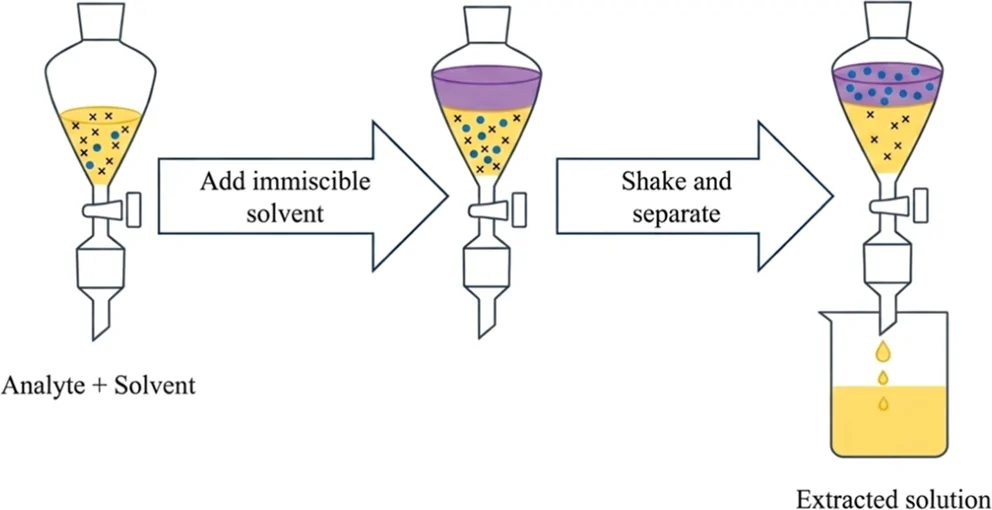
Conventional LLE using two immiscible liquids.

Liquid-phase microextraction techniques represent a significant advancement in sample preparation techniques,^85^ offering a miniaturized alternative to classical LLE.^61^ The miniaturized techniques use small volumes of organic solvents, typically ranging from 1.00 to 100.00 μL, to extract analytes from sample matrices.^86^ Their fundamental principle involves the use of low volumes of immiscible solvents, which act as an acceptor phase for the extraction of compounds from the aqueous phase (donor phase).^87^ The commonly used liquid phase microextraction techniques include SDME, HF-LPME, and DLLME.^87–90^ Several researchers have reported typical extraction performance of LLE and its miniaturized versions on selected pharmaceuticals from different environmental samples in terms of recovery and enrichment factors. Table 2 summarizes recoveries and enrichment factors for several pharmaceuticals using LLE and its common miniaturized versions, as reported in selected studies. The values have been reproduced from the cited studies and may vary owing to differences in analytes, sample matrices, and experimental conditions.

**Table 2:** Recovery and Enrichment Factors for LLE and Selected Miniaturized LLE Techniques^a^

Extraction technique	Nature of sample	Targeted pharmaceutical(s)	Solvent(s) used	Reported recovery (%)	Enrichment factors	LOD (μg/L)	Reference
LLE	Wastewater	Imatinib, doxorubicin, etoposide, megestrol, prednisone, bicalutamide, capecitabine, cyclophosphamide, ifosfamide, and mycophenolic acid	Dichloromethane and acetonitrile	74–94	NR	0.0002–0.08	^84^
	Seawater and sediments	Oxytetracycline, florfenicol, and flumequine	Methanol and acetonitrile	63–120	NR	0.3–1.5	^91^
	Wastewater	Amitriptyline	Tetrabutylammonium bromide and tetrabutylammonium chloride	92–100	NR	NR	^83^
SDME	Surface water	Ranitidine	Dichloromethane and water	98.3–101.3	45	0.0094	^92^
	Surface water and groundwater	Enrofloxacin, ciprofloxacin, and ofloxacin	Dichloromethane and toluene	90.0–100.3	6–40	10.1–55.3	^93^
	Surface water and wastewater	Naproxen, diclofenac, and ibuprofen	Methanol and toluene	65.2–123.7	70–89	0.03–0.2	^94^
HF-LPME	Wastewater	Ibuprofen, naproxen, and ketoprofen	Octanol	89.2–108.2	133–272	0.001–0.002	^95^
	Wastewater and surface water	Emtricitabine, tenofovir disoproxil, and efavirenz	Dihexyl ether and water	86–111	24–111	0.009–0.16	^96^
	Surface water	Danofloxacin, norfloxacin, enrofloxacin, and ciprofloxacin	Methanol and water	9.4–24.5	NR	0.1–10	^97^
	Wastewater	Salicylic acid, ibuprofen, and diclofenac	Dihexyl ether and water	52–100	NR	0.02–0.3	^98^
DLLME	Wastewater	Ofloxacin, norfloxacin, enrofloxacin, and lomefloxacin	Methanol and tetrachloroethane	82.7–110.9	32–134	0.14–0.81	^99^
	Seawater and seafood	Ofloxacin, ciprofloxacin, enrofloxacin moxifloxacin, and pefloxacin	Acetonitrile, methanol, and hexane	62.3–111.8	11.3–146	0.90–29.20	^100^
	Wastewater	Sulfamethoxazole and ciprofloxacin	Methanol and water	79.13–120.43	NR	0.78–1.58	^101^

a Abbreviations: limit of detection (LOD), liquid–liquid extraction (LLE), single-drop microextraction (SDME), hollow fiber-liquid phase microextraction (HF-LPME), dispersive liquid–liquid microextraction (DLLME), NR—not reported.

#### 3.1.1 Conventional Liquid–Liquid Extraction

LLE is a fundamental separation process based on the differential distribution of a solute between two immiscible solvents. The efficiency of this method relies on the partition coefficient, which dictates how compounds partition between two phases, typically an aqueous phase and an organic solvent, based on their relative solubility in each.^83^ LLE remains a cornerstone of analytical chemistry due to its versatility in isolating a wide array of organic compounds from aqueous environments and complex matrixes.^84^ In environmental analysis, LLE has served as a primary sample preparation technique for decades. It provides a robust framework for extracting active ingredients and metabolites from different sample matrices and is recognized as a standard protocol in environmental applications.^82,102^ Several researchers have used LLE as a method of choice for environmental monitoring of pharmaceuticals and related products.

In addition to conventional LLE, solvent flotation (SF) extraction has emerged as a promising LLE-based variant that combines solvent extraction with flotation, enabling analyte enrichment while substantially reducing solvent consumption.^103^ The technique is attractive for trace-level environmental analysis because it offers efficient preconcentration with lower volumes of organic solvents than conventional LLE.^104^ Common extraction solvents employed in LLE for pharmaceutical analysis include chlorinated solvents (e.g., dichloromethane and tetrachloroethane) and organic solvents (e.g., methanol, acetonitrile, and hexane), as well as modern ionic liquids and deep eutectic solvents as greener alternatives.^105,106^

Table 2 indicates the performance of conventional LLE in the preconcentration of selected pharmaceuticals from wastewater.^83,91^ Recoveries in the range 74–94% have been reported for a wide range of pharmaceuticals, including imatinib, doxorubicin, etoposide, megestrol, prednisone, bicalutamide, capecitabine, cyclophosphamide, ifosfamide, and mycophenolic acid in wastewater.^102^ By employing special ionic liquids as extraction solvents, Gouveia et al.^84^ managed to achieve 100% maximum recovery of amitriptyline from wastewater using conventional LLE. This clearly demonstrates the versatility of LLE in extracting and preconcentrating compounds with varied properties. LLE was also successfully used to extract and preconcentrate antibiotics used in aquaculture (oxytetracycline, florfenicol, and flumequine) from seawater and sediments with recoveries of 63–120%.^91^ LLE can be easily adjusted to meet required extraction goals by changing solvents, using multiple solvents, and repeated extractions.

In terms of practical and sustainability-related features (Table 1), conventional LLE is moderately simple as the procedure is straightforward but involves multiple handling steps, such as phase separation and solvent transfer. Its rapidness is generally low, mainly due to longer equilibration times and repeated extraction cycles that are often required to improve recovery. The selectivity is low since extraction is primarily governed by solubility and partitioning rather than specific molecular interactions, which may result in coextraction of matrix components. However, LLE demonstrates high versatility (broad applicability) as solvent choice can be easily adjusted to target pharmaceuticals with diverse physicochemical properties across wastewater, seawater, and sediment matrices.^60^ From a sustainability perspective, LLE scores low in greenness owing to the use of relatively large volumes of often hazardous organic solvents, usually >20.0 mL per extraction run. Similarly, reusability is low since solvents are typically single-use and the method does not involve reusable extraction media.^59^

#### 3.1.2 Single-Drop Microextraction

SDME is a miniaturized LLE technique that involves suspending a drop of water-immiscible organic extracting solvent (approximately 1.0–10.0 μL) from a syringe into the liquid or gaseous sample medium.^15^ SDME, also referred to as liquid–liquid microextraction (LLME), involves the distribution of analytes between an aqueous sample and a small droplet of solvent suspended at the tip of a micro syringe needle.^86^ The SDME technique can be divided into two main categories based on the mode of operation: direct-immersion single-drop microextraction (DI-SDME) (Figure 4a) and headspace single-drop microextraction (HS-SDME).^39,88^ Researchers have reported good recoveries of pharmaceuticals from surface water samples (Table 2), using SDME as the pre-concentration technique.^92–94^ Kiszkiel-Taudul et al.^92^ reported up to 101.3% recovery and 45-fold enrichment of ranitidine from surface water using SDME. Recoveries of 88.1–123.7% and 70–89 enrichment factors have been reported using SDME for naproxen, diclofenac, and ibuprofen in wastewater and surface water samples.^94^ Relatively high recoveries (90.0–100.3%) were reported for fluoroquinolone antibiotics: enrofloxacin, ciprofloxacin, and ofloxacin in groundwater using SDME.^93^ SDME is preferred over traditional extraction methods because it is simple, environmentally friendly, rapid, and highly efficient.^107^ In addition, the method offers advantages, including minimal solvent consumption, cost-effectiveness, and compatibility with various analytical techniques.^87,108^

**Figure 4:**
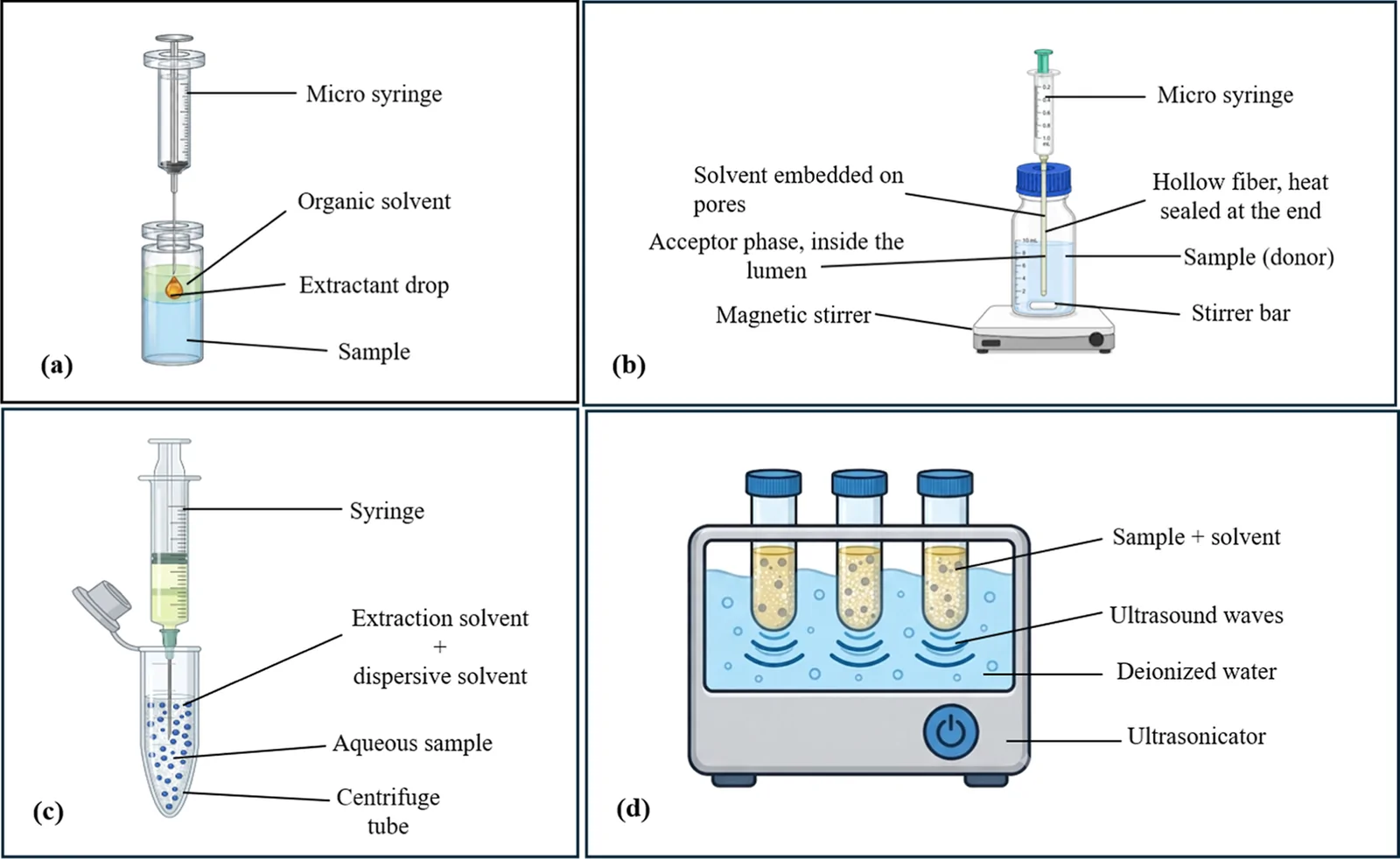
Selected liquid phase-based miniaturized techniques: (a) DI-SDME setup. Reprinted from ref 122 Copyright 2018, with permission from Elsevier. (b) HF-LPME setup. Reprinted from ref 123 Copyright 2022, with permission from Elsevier. (c) DLLME. (d) UA-LLME.

From the practicality perspective outlined in Table 1, SDME demonstrates high simplicity as it requires minimal equipment and operational steps compared to conventional LLE.^61^ Its rapidness is high since equilibrium is typically reached quickly due to the small extraction volume and short diffusion distances. In terms of selectivity, SDME is moderate as extraction is governed mainly by solvent–analyte affinity; while it can effectively concentrate target pharmaceuticals, matrix interferences may still occur in complex wastewater samples. The versatility of SDME is considered limited because the small droplet volume and solvent stability constraints may restrict its application to certain analytes and matrices. On the other hand, SDME scores high in greenness due to its extremely low solvent consumption (microliter scale), making it environmentally favorable compared to conventional LLE. However, reusability is low as each solvent droplet is single-use and cannot be recycled without compromising analytical performance.^62^ On the contrary, SDME is not an exhaustive extraction technique, meaning only a small fraction of the analyte is extracted and pre-concentrated for analysis.^25^ The technique has been widely employed as a green microextraction method for trace analysis in chemical, biological, food, environmental, clinical, pharmaceutical, and forensic analysis.^86^

#### 3.1.3 Hollow-Fiber Liquid-Phase Microextraction

HF-LPME is usually based on the use of disposable porous polymeric hollow fibers that are filled with a small amount of extracting solvent (acceptor phase).^88,108^ To extract the target analytes, the fiber is immersed in the aqueous sample solution (donor phase).^14,88^ HF-LPME can operate in both two-phase and three-phase device modes using donor–acceptor interactions^22^ as depicted in Figure 4b. This configuration allows the technique to reach high extraction efficiency and enables the detection and quantification of a wide class of analytes at low levels.^14,97^ HF-LPME has been applied for the extraction and pre-concentration of various pharmaceutical compounds from environmental samples.^81,109^ Es’haghi et al.^95^ and Mlunguza et al.^96^ successfully extracted and pre-concentrated non-steroidal anti-inflammatory drugs (NSAIDs), such as ibuprofen, naproxen, and ketoprofen, in wastewater and antiretroviral drugs (ARVs) such as emtricitabine, tenofovir, disoproxil, and efavirenz in surface water and wastewater using HF-LPME (Table 2). Up to 108.2% recovery and 272-fold enrichment were reported for NSAIDS using HF-LPME.^95^ ARVs in surface and wastewater had up to 111% recovery and 111 times enrichment using the same technique.^96^ The reported high recoveries and huge enrichment factors have been linked to the technique’s high selectivity and sensitivity.^96,98^ HF-LPME offers several advantages, including the requirement of only a small sample volume, making it cost-effective and environmentally friendly.^14^

In line with the practical attributes presented in Table 1, HF-LPME demonstrates moderate simplicity as it requires preparation and impregnation of the hollow fiber but does not involve complex instrumentation. Its rapidness is moderate since equilibrium may require controlled extraction times depending on analyte diffusion through the fiber membrane.^63^ The technique exhibits high selectivity, particularly in three-phase systems, where pH control allows selective transport of ionizable pharmaceuticals across the membrane. The versatility is moderate as HF-LPME performs well for many polar and semipolar pharmaceuticals but may require system adjustments for highly hydrophobic compounds.^64^ In terms of sustainability, HF-LPME scores high in greenness due to the use of microliter volumes of organic solvent.^63^ However, reusability is generally low since hollow fibers are often treated as disposable to avoid cross-contamination and performance loss. Like other microextraction techniques, HF-LPME is simple, environmentally friendly, rapid, and highly efficient compared with traditional extraction methods.^90,107,109^

Despite its strong performance, HF-LPME requires careful optimization to ensure the reproducibility and high extraction efficiency. Critical parameters that must be optimized include donor and acceptor phase pH (to control analyte ionization and transport), extraction time (to achieve equilibrium), stirring rate (to enhance mass transfer), organic solvent selection (to ensure membrane stability and analyte affinity), and salt addition (to improve partitioning through salting-out effects).^110^ In three-phase systems, maintaining appropriate pH gradients between donor and acceptor phases is particularly important for facilitating selective analyte migration.^111^ Researchers often address these issues through a systematic experimental design and response surface optimization to balance selectivity, enrichment, and extraction time. Overall, HF-LPME remains a highly attractive microextraction technique for trace pharmaceutical analysis in environmental matrices, particularly when high enrichment and selectivity are required if extraction conditions are carefully controlled and optimized.

#### 3.1.4 Dispersive Liquid–Liquid Microextraction

DLLME has emerged as the most widely used form of liquid-phase microextraction.^88^ DLLME is based on the initial fast injection of a suitable mixture of two solvents—an extraction solvent and a dispersive solvent into an aqueous sample solution with the assistance of a syringe (Figure 4c), followed by the formation of a cloudy solution that contains droplets of the extraction solvent dispersed into the sample.^88^ After phase separation due to the difference in density of the two phases (e.g., by centrifugation), the extraction phase can be removed and analyzed.^88^ In the conventional form of DLLME, the extraction phase is accumulated at the bottom of the extraction container.^88^ The technique has been widely applied to preconcentrate and separate organic and inorganic compounds, including pharmaceuticals in environmental samples (Table 2). The performance of DLLME as an extraction and preconcentration technique has been reported in previous studies.^99,101^ Yan et al.^99^ indicated that DLLME achieved up to 110.9% recovery of fluoroquinolones in pharmaceutical wastewater, with enrichment factors reaching 134-fold. Different researchers^101^ also achieved maximum recoveries of 120.4% and 111.5% for sulfamethoxazole and ciprofloxacin in wastewater, respectively, using DLLME.

DLLME was also used in the extraction and preconcentration of pharmaceuticals from seawater and seafood samples. In that regard, Lou et al.^100^ managed to recover ofloxacin, ciprofloxacin, enrofloxacin, moxifloxacin, and pefloxacin from seafood with recovery values ranging from 62.3 to 111.8%. DLLME also recorded exceptionally high enrichment of these antibiotics, reaching a maximum of 146-fold.^100^ This previous work clearly shows that DLLME is a novel sample preparation technique offering several advantages, such as high recovery, high extraction efficiency, stability, and sensitivity.^112^

As per practical aspects, DLLME exhibits moderate simplicity as it involves straightforward solvent injection and centrifugation steps, although careful handling of solvent ratios is required.^65^ Its most outstanding feature is very high rapidness since dispersion and mass transfer occur almost instantly, significantly reducing extraction time compared to conventional LLE.^66^ In terms of selectivity, DLLME is moderate as extraction is mainly governed by solvent affinity and may not fully discriminate against co-extracted matrix components. The versatility is moderate as DLLME performs well for many semipolar pharmaceuticals but may require pH adjustment for strongly ionized compounds. Its reusability is low since the extraction solvent is consumed in each run and cannot be recycled without affecting performance.

Various modifications and assisted variants of DLLME have been developed to enhance the extraction efficiency. These include ultrasound-assisted DLLME (UA-DLLME), vortex-assisted DLLME, and magnetic stirring-assisted dispersive liquid–liquid microextraction (MSA-DLLME).^87,113^ Ionic liquids have also been successfully incorporated into DLLME techniques, with ionic liquid-based dispersive liquid–liquid microextraction (IL-DLLME) being developed as a green solvent alternative.^114,115^

Despite its many advantages, DLLME has several notable drawbacks in environmental monitoring applications. One major limitation is the continued reliance on toxic and volatile organic solvents, particularly chlorinated extraction solvents, which reduces the overall “greenness” of the technique despite its low solvent volumes.^116^ In addition, DLLME generally offers limited selectivity, making it susceptible to matrix effects when applied to complex environmental samples such as wastewater, where co-extraction of interfering compounds can compromise analytical accuracy.^99^ Furthermore, DLLME is less suitable for highly polar or strongly ionized pollutants as these compounds tend to remain in the aqueous phase.^101^ Overcoming this often requires pH adjustment or derivatization, which increases method complexity and may affect reproducibility.

#### 3.1.5 Emerging Miniaturized Liquid–Liquid Extraction Techniques

Beyond the classical LLE miniaturized techniques, several specialized and emerging methods have been developed to address specific analytical challenges and improve the environmental sustainability. Salting-out-assisted liquid–liquid extraction (SALLE) represents one such advancement that has been applied to pharmaceutical extraction from environmental samples.^38^ The salting-out approach combines the advantages of dispersive liquid microextraction with enhanced extraction efficiency, as demonstrated by the development of salting-out-enhanced ionic liquid microextraction based on dual-role solvents, which achieve higher extraction efficiency for drugs and phenolic contaminants with a wide range of polarities in aqueous samples.^112^ However, the need for ionic liquids might increase the overall cost of the method.

Additionally, ultrasound-assisted liquid–liquid microextraction (UA-LLME) has been developed as a variant that enhances extraction efficiency through mechanical assistance (Figure 4d).^38^

A significant trend in modern LPME involves the use of ionic liquids as green extraction solvents. Ionic liquids have been rapidly developed as environmentally friendly acceptor phases for various microextraction techniques in sample preparation, including liquid-phase microextraction, single-drop liquid-phase microextraction, solid-phase microextraction, dispersive liquid-phase microextraction, and cold-induced aggregation microextraction.^117^ These applications have expanded to include ionic liquid-based liquid–liquid microextraction (IL-LLME), ionic liquid-based single-drop microextraction (IL-SDME), ionic liquid-based dispersive liquid–liquid microextraction (IL-DLLME), in situ solvent formation microextraction (ISFME), and temperature-controlled ionic liquid dispersive liquid-phase microextraction (TILDLME).^114^

Deep eutectic solvents (DESs) represent another emerging class of green solvents that have been successfully applied in LPME techniques. Pharmaceutical preservatives, such as parabens, have also been used as model compounds in some studies to demonstrate the applicability of these solvents. Hydrophobic deep eutectic solvents formed in situ by DL-menthol and decanoic acid have been used as extraction solvents in deep eutectic solvent-liquid–liquid microextraction methods for the determination of these parabens in aqueous samples.^118^ Polymeric deep eutectic solvents composed of DL-menthol and polyethylene glycol with different molecular weights have been prepared and used as extraction solvents, achieving good recoveries in the range of 87.9–107.8% for paraben extraction.^119^ Deep eutectic solvent-based techniques have also been specifically applied for antibiotic drug extraction, including deep eutectic solvent liquid–liquid microextraction (DES-LLME) for the determination of fluoroquinolones in milk, honey, and water samples and salting-out-assisted dispersive liquid–liquid microextraction based on deep eutectic solvents for the determination of levofloxacin in urine samples.^120^ The integration of hydrophobic deep eutectic solvents with shaker-assisted liquid–liquid microextraction (hDES-SA-LLME) represents a notable recent development that combines the benefits of green solvents with mechanical assistance for enhanced extraction.^38^ Additionally, novel supramolecular deep eutectic solvents (SUPRADES) have been developed and utilized as disperser/extraction solvent mixtures in dispersive liquid–liquid microextraction for various pharmaceutical compounds, including tetracyclines and sulfonamides in environmental waters.^121^

Despite these improvements, no single technique is capable of efficiently extracting all classes of pharmaceuticals across the diverse environmental matrixes. Instead, most techniques tend to perform best under specific conditions and for a selected group of analytes. As a result, the choice of an appropriate extraction method should be made on a case-to-case basis. A holistic approach is therefore required, one that carefully considers factors such as analyte properties, matrix complexity, and method compatibility to achieve an optimal extraction efficiency and reliable recovery of the target pharmaceuticals.

### 3.2 Solid-Phase Extraction (SPE) and Miniaturized Versions

The most common sample isolation and preconcentration technique for the extraction of pharmaceuticals in water samples is SPE.^82^ SPE is a sample preparation technique that uses a solid stationary phase to separate compounds based on differences in their affinities for the stationary phase and the mobile phase.^124^ The principle involves selectively retaining either the target analytes or unwanted interferences on the solid phase, allowing for the collection of the purified sample.^16^ The analytical method development using SPE in environmental analysis allows the use of large sample volumes, around 50 to 1000 mL, and thus increases the concentration by a factor of 100 to 5000, making it possible to perform qualitative and quantitative analysis at trace levels.^125^ The conventional SPE technique requires at least four steps for sample preparation: (1) sorbent conditioning, (2) sample loading, (3) washing (to remove interferers and matrix concomitants) and, (4) elution of the analytes,^125^ as depicted in Figure 5.

**Figure 5:**
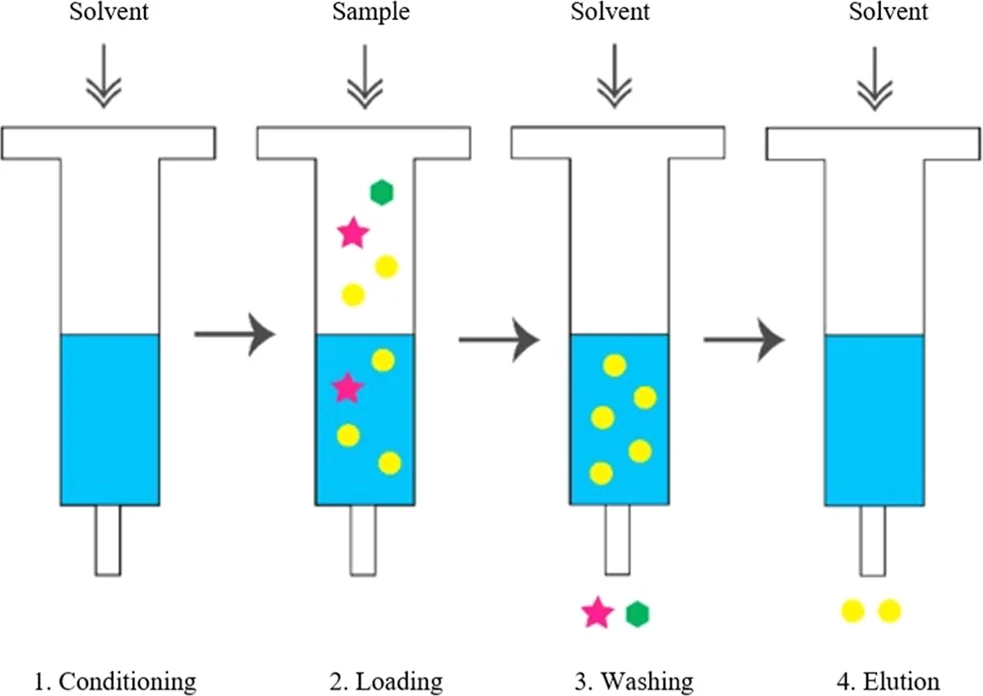
Conventional SPE technique. Reprinted from ref 126 Copyright 2021, with permission from Elsevier.

The development of new sorbent materials has led to several modifications of SPE methods in recent years. Modern SPE techniques are now based on miniaturization and automation.^125^ Different SPE microextraction techniques have been proposed as efficient alternatives to the classical SPE technique.^88^ These miniaturized sample preparation techniques have shown improved extraction of pharmaceuticals from environmental samples compared with conventional SPE, as indicated by pharmaceutical recovery values reported in previous studies.^127,128^ Table 3 summarizes pharmaceutical recovery results from environmental samples using SPE and its commonly used miniaturized variants, as reported by selected researchers. The reported values were directly reproduced from the cited studies and may vary because of differences in analytes, sample matrices, and experimental conditions.

**Table 3:** Recovery Values for SPE and Selected Miniaturized SPE Techniques^a^

Extraction technique	Nature of sample	Targeted pharmaceutical (s)	Sorbent material	Reported recovery (%)	LOD (μg/L)	Reference
SPE	Surface water and seawater	Efavirenz, ibuprofen, naproxen, sulfamethoxazole, and trimethoprim	HLB	56–117	0.1–0.8	^129^
	Wastewater	Salicylic acid, ibuprofen, fenoprofen, diclofenac, and naproxen	HLB, oasis MAX, OASIS WAX, and MIP	70–85	0.025–0.2	^130^
	Wastewater	Acetaminophen, albuterol, allopurinol, amitriptyline, brompheniramine, carbamazepine, carisoprodol, ciclopirox, diazepam, fenofibrate, metoprolol, primidone, and terbinafine	HLB, C18, MCAX, MCX, strata X, and strata XC	9–109	0.005–0.05	^131^
	Spiked water	Carbamazepine, camphor, benzophenone-1, and ibuprofen	HLB	82.5–90.0	1.6–2.9	^132^
SPME	Wastewater	Sulfaguanidine, sulfacetamide, sulfadiazine, and sulfamethoxazole	MCX, PDMS and CW/DVB	29–229	0.028–0.09	^128^
	Wastewater	Carbamazepine, fluoxetine, sertraline, and paroxetine	PDMS and C18	71.0–87.5	0.002–0.01	^133^
	Wastewater	Estrone; 17-β-estradiol; 17-α-ethinyl estradiol, clofibric acid, and carbamazepine	Polyacrylate fiber	89–105	0.075–0.75	^134^
MSPE	Wastewater	Ketoprofen, diclofenac sodium, and ibuprofen	Magnetite	48.9–110.3	0.5–0.4	^135^
	Wastewater	Sulfapyridine, sulfamethoxypyridazine, sulfamethoxazole, and sulfamonomethoxine	Fe_2_O_3_–Fe/MMC	75.40–122.12	0.102–0.122	^127^
	Wastewater and river water	Atenolol, metoprolol, esmolol, pindolol, and arotinolol	Mag-MWCNTs	82.9–95.6	0.0005–0.0015	^136^
MSPDE	Sewage sludge	Propranolol, losartan, irbesartan, telmisartan, and valsartan, flecainide, dronedarone, and amiodarone	C18	82–124	0.002–0.01	^137^
	Seafood and seawater	Trimethoprim, sulfamethoxazole, sulfadiazine, and sulfamethazine	C18, PSA, and florisil	27.2–58.6	0.03–0.3	^138^
	Wastewater	Acetaminophen, naproxen, diclofenac, and ibuprofen	Magnetite, silica, and C8	94–100	1.0–2.0	^139^
SBSE	Wastewater	Amitriptyline, azithromycin, desipramine, diazepam, donepezil, escitalopram, fluoxetine, haloperidol, imatinib, clomipramine, loperamide, loratadine, metoprolol, promethazine, propranolol, raloxifene, risperidone, selegiline, sertraline, tramadol, triclosan, venlafaxine, verapamil, ziprasidone, bromazepam, carbamazepine, and clonazepam	PDMS	25–85	0.002–0.005	^140^
	Seafood	Enoxacin, norfloxacin, and ciprofloxacin	UiO-66-(OH)_2_	74.8–115.8	0.0005–0.0008	^141^
	Spiked water	Carbamazepine, β estradiol, 3-(4-methylbenzylidene) camphor, benzophenone-1, and ibuprofen	PDMS	71.4–83.2	1.6–2.9	^132^

a Abbreviations: limit of detection (LOD), solid-phase extraction (SPE), solid-phase microextraction (SPME), magnetic solid-phase extraction (MSPE), matrix solid-phase dispersion extraction (MSPDE), stir-bar sorptive extraction (SBSE), hydrophobic–lipophilic balance (HLB), Oasis MAX (strong anion exchange), Oasis WAX (weak anion exchange), molecularly imprinted polymer (MIP), polydimethylsiloxane (PDMS), carbowax/divinylbenzene (CW/DVB), magnetic mesoporous carbon (Fe_2_O_3_–Fe/MMC), magnetic multiwalled carbon nanotubes (Mag-MWCNTs), weak anion exchanger sorbent (PSA), and hydroxyl-functionalized zirconium (UiO-66-(OH)_2_).

#### 3.2.1 Conventional Solid-Phase Extraction

Conventional SPE is regarded as a relatively cheap and reliable approach that can be applied to a variety of target analytes. Several researchers make use of this approach in extracting and preconcentrating a variety of analytes, including pharmaceuticals, in environmental monitoring work.^129–131,142^ SPE has been successfully used in extracting and preconcentrating antiretroviral drugs and nonsteroidal anti-inflammatory drugs (NSAIDs) in surface and wastewater,^129^ as well as anticonvulsant and analgesics in wastewater.^131^ Netshithothole et al.^129^ achieved a maximum recovery of 83% on an antiretroviral drug (efavirenz) in wastewater sludge and 117% on naproxen, an NSAID, in wastewater (Table 3). Recoveries in the range 70–85% have been reported using conventional SPE for salicylic acid, ibuprofen, fenoprofen, diclofenac, and naproxen.^130^

Bisceglia et al.^131^ reported recoveries for different pharmaceuticals from wastewater in the range 9–109% using conventional SPE. The recovery of pharmaceuticals using conventional SPE seems to be analyte-specific, with some pharmaceuticals having low recoveries (9% for acetaminophen in influent) and others having relatively higher recoveries (109% for brompheniramine in the same influent) within the same experiment.^131^ These differences in extraction efficiency on different analytes can be attributed to the selectivity of the sorbent employed.^142^ Conventional broad-spectrum sorbents, such as hydrophilic–lipophilic balanced (HLB) polymeric sorbents (e.g., Oasis HLB), are intentionally designed to provide broad applicability across a wide range of pharmaceutical compounds. While this broad-spectrum nature may result in lower selectivity for certain pharmaceutical classes, it is a significant advantage for non-target screening and multi-residue analysis in complex environmental samples. In contrast, more selective sorbents, including ion-exchange, mixed-mode, and molecularly imprinted polymers (MIPs), can substantially improve selectivity and extraction efficiency for specific target analytes. Consequently, the overall selectivity of SPE depends largely on the sorbent chemistry and the analytical objective rather than the extraction technique itself.

Furthermore, the adaptability of the SPE can be significantly enhanced through the design and synthesis of tailored sorbents. For instance, molecularly imprinted polymers (MIPs) have been developed as highly selective SPE sorbents that can target specific pharmaceuticals based on the analytical need.^143,144^ Such materials not only improve selectivity and extraction efficiency but also offer improved reusability compared to conventional cartridges as they are generally more resistant to fouling and degradation. However, the development of new SPE sorbent materials has led to numerous modifications, giving rise to miniaturized and automated approaches.^125,142^

Based on the practicality summarized in Table 1, conventional SPE demonstrates moderate simplicity as it involves multiple steps such as conditioning, sample loading, washing, and elution but remains straightforward and widely standardized.^69^ Its rapidness is moderate since sample loading and elution require controlled flow rates and can be time-consuming for large sample volumes. In contrast to LLE, SPE exhibits high selectivity, particularly when appropriate sorbents (e.g., reversed-phase, ion-exchange, or mixed-mode) are selected to match analyte properties.^145^ The technique also shows broad versatility as non-selective sorbent materials allow the extraction of pharmaceuticals with diverse physicochemical characteristics across various environmental matrices.^70^ Regarding sustainability, SPE scores low in greenness, mainly due to the use of organic solvents for conditioning and elution, although solvent volumes are generally lower than those in conventional LLE. Reusability varies depending on the sorbent type; while many commercial cartridges are limited in reuse due to fouling and carryover, advanced sorbents such as MIPs can be reused multiple times without a significant loss of performance.^59^

Overall, conventional SPE remains one of the most dependable and adaptable sample preparation techniques in environmental pharmaceutical analysis, particularly when a high selectivity and broad applicability are required.

#### 3.2.2 Solid-Phase Microextraction

The SPME technique is a fast, largely solvent-free extraction technique for sampling, cleaning up, and preconcentration of analytes.^146,147^ The technique integrates sampling, extraction, preconcentration, and injection into one analytical system, which is simple and efficient.^148^ The entire sample preparation can be solvent-free for gas chromatography, while minimal solvent is required for reconstitution and desorption for liquid chromatography. SPME can be categorized into two forms: headspace SPME (HS-SPME) and direct immersion SPME (DI-SPME). In HS-SPME, the technique consists of a fiber exposed to the headspace above the sample to extract volatile or semivolatile compounds.^14,15^ In DI-SPME, the sample is in direct contact with the extraction fiber, allowing efficient uptake of non-volatile analytes from aqueous matrices through partitioning between the sample and the fiber coating (Figure 6a).^149,150^

**Figure 6:**
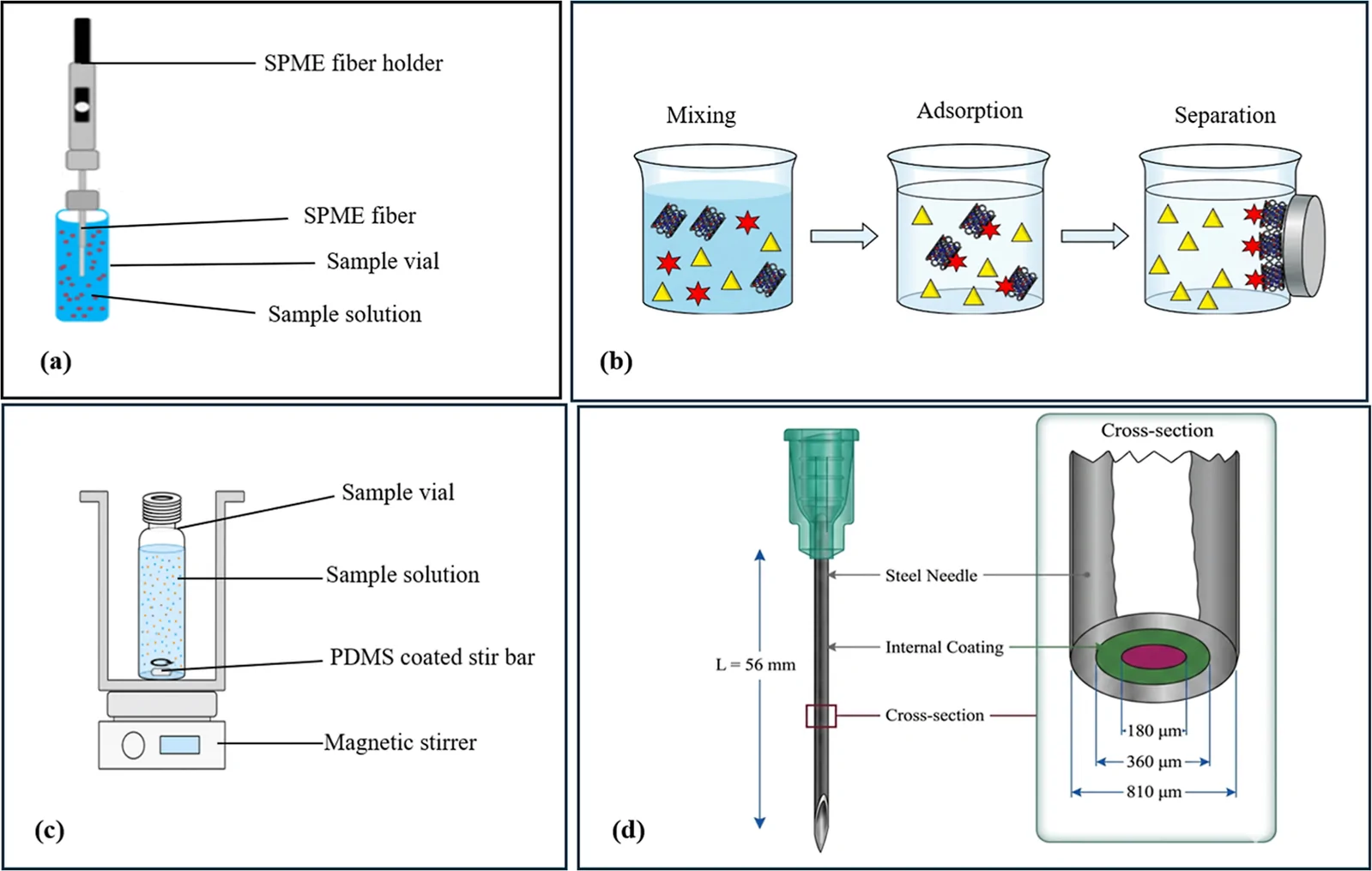
Selected solid-phase-based miniaturized techniques: (a) basic DI-SPME setup. Reprinted with permission from ref 166 Copyright 2020 American Chemical Society. (b) Schematic illustration of the MSPE procedure. Reprinted from ref 153 Copyright 2020, with permission from Elsevier. (c) SBSE and (d) SPDE.

SPME has been used extensively for environmental applications.^151^ Several researchers have successfully extracted and preconcentrated various pharmaceuticals, including antibiotics and hormones, from contaminated wastewater using SPME.^128,133,134^ Extraction efficiencies of up to 229% have been reported as recovery for sulfonamide antibiotics, specifically sulfaguanidine, sulfacetamide, sulfadiazine, and sulfamethoxazole in wastewater.^128^ The reported recoveries suggest effective extraction of sulfonamides under the optimized experimental conditions adopted in the respective study. Antoniou et al.^134^ also reported recoveries approaching or exceeding 100% for hormones from wastewater using SPME, with values ranging from 89 to 105%. However, relatively lower recoveries (71.0–87.5%) have been reported for other pharmaceuticals including carbamazepine, fluoxetine, sertraline, and paroxetine in wastewater.^133^ Lower recoveries for compounds, such as carbamazepine, can be attributed to polar functional groups, which promote stronger interactions with the aqueous matrix and reduce partitioning onto the SPME fiber.

In line with the practical aspects outlined in Table 1, SPME demonstrates moderate simplicity as the procedure is operationally straightforward but requires careful handling of fragile fibers and optimization of extraction conditions.^72^ Its rapidness is moderate since equilibrium times vary with analyte diffusion and coating type. One of the major strengths of SPME is its high selectivity, which arises from the availability of different fiber coatings tailored to specific analyte classes. The versatility is moderate as SPME performs well for a broad range of volatile, semivolatile, and some non-volatile pharmaceuticals, although highly polar compounds may require specialized coatings. From a sustainability perspective, SPME scores high in greenness because it is essentially solvent-free.^71^ Additionally, reusability is high as SPME fibers can be reused multiple times under controlled conditions, making the technique cost-effective and environmentally favorable over repeated analyses.^72^ SPME remains a powerful and environmentally sustainable technique for pharmaceutical monitoring in environmental matrices, particularly when selectivity and solvent-free operations are key considerations.

#### 3.2.3 Magnetic Solid-Phase Extraction

MSPE, as a new type of SPE, has received considerable interest in recent years.^152^ The technique is recognized among typical examples of miniaturized sample preparation techniques that have been proposed as efficient alternatives to classical extraction techniques.^88^ MSPE involves the use of magnetic nanoparticles, acting as adsorbents to selectively bind and isolate target analytes from a liquid sample.^153^ Once adsorption is complete, an external magnetic field is applied to easily separate the analyte-bound nanoparticles from the matrix without the need for traditional filtration or centrifugation steps, as illustrated in Figure 6b. MSPE has been employed in the extraction of various contaminants of emerging concern, including pharmaceuticals from the environment.^127,154^ Recent studies on environmental monitoring of pharmaceuticals have seen successful extraction and preconcentration of antibiotics such as sulfapyridine, sulfamethoxypyridazine, sulfamethoxazole, and sulfamonomethoxine, with recoveries in the range 75.4–122.1% and enrichment factors reaching a maximum of 49-fold.^127^

Commonly reported NSAIDs such as ketoprofen, diclofenac, and ibuprofen have also been successfully concentrated with this technique, giving recoveries of up to 48.9–110.3% and an 11 times enrichment factor.^135^ These findings indicate that MSPE possesses excellent recovery and enrichment for a variety of pharmaceuticals. This can be attributed to the fact that MSPE utilizes various types of magnetic adsorbents, with the magnetic properties of the sorbent materials allowing for easy separation and recovery.^154^ Magnetic adsorbents use external magnetic fields, eliminating the need for centrifugation or filtration steps that are typically required in conventional SPE procedures. This makes MSPE a simple, rapid, and less costly technique in terms of the use of solvents.^18^ The technique offers several practical advantages over traditional extraction methods, particularly in terms of its operational efficiency and environmental impact. The recent literature demonstrates that MSPE and its variations, including MSPE-LLME combinations, are increasingly utilized in pharmaceutical extraction studies, representing a significant portion of miniaturized extraction techniques employed in environmental sample analysis.^38^

From a practical perspective (Table 1), MSPE is moderately simple as the procedure involves sorbent dispersion and magnetic retrieval but may require the synthesis or preparation of specialized magnetic materials.^73^ Its rapidness is high, mainly due to the rapid magnetic separation step that significantly shortens the extraction time compared to cartridge-based SPE. MSPE exhibits high selectivity, particularly when functionalized magnetic sorbents are designed to target specific pharmaceuticals. The versatility is moderate since different magnetic nanomaterials can be engineered to accommodate various analytes and environmental matrices.^74^ In terms of sustainability, MSPE scores moderate in greenness, while solvent consumption is reduced, and some preparation steps and sorbent synthesis may involve chemical reagents. Finally, reusability is moderate as magnetic sorbents can often be regenerated and reused multiple times, although performance may gradually decline due to fouling or loss of active sites. MSPE stands out as a highly promising miniaturized extraction technique, particularly when rapid separation, high selectivity, and operational convenience are key considerations in the pharmaceutical monitoring of environmental samples.

#### 3.2.4 Matrix Solid-Phase Dispersion Extraction

MSPDE is a sorbent-based extraction technique,^155^ involving mixing samples, mostly solids, with a dispersant sorbent material to achieve complete sample disruption and isolate the analytes.^138^ The analytes are then eluted using an appropriate solvent.^156^ MSPDE has also emerged as one of the novel extraction techniques based on miniaturization and automation.^157,158^ The MSPDE technique combines extraction and purification into a single step, facilitating the analysis of contaminants in environmental, food, and other complex matrices.^137^ Several researchers have reported the extraction efficiencies of MSPDE for a variety of pharmaceuticals from different environmental matrices, including wastewater, sewage sludge, seafood, and seawater.^137–139^ The analgesics, acetaminophen and NSAIDS: naproxen, diclofenac, and ibuprofen, were recovered in wastewater samples at relatively high recoveries (94–100%) using MSPDE.^139^ Castrol et al.^137^ also reported relatively high recoveries (82–124%) of antihypertensive drugs (propranolol, losartan, irbesartan, telmisartan, and valsartan) and antiarrhythmic drugs (flecainide, dronedarone, and amiodarone) in sewage sludge. These results indicate the versatility of MSPDE in extracting different types of pharmaceuticals. This can mainly be because MSPDE allows the use of different solvents depending on the target analyte’s properties.

Limited recoveries (27.2–58.6%) of antibiotics, namely, trimethoprim, sulfamethoxazole, sulfadiazine, and sulfamethazine, have been reported in seafood and seawater using MSPDE.^138^ This could be because of the complexity of sea-based samples, which requires thorough optimization. MSPDE provides exhaustive extractions and can substantially reduce matrix effects through the simultaneous disruption, extraction, and cleanup of the sample matrix, thereby improving analytical performance. However, the extent of matrix interference remains dependent on the sample matrix, target analytes, and extraction conditions.^137^ The method requires a moderate consumption of organic solvents and does not use dedicated instrumentation. However, to improve extraction efficiency in MSPDE, there is a need to choose the most appropriate dispersant–sorbent material and a solvent(s) system that suits the target analyte.^156^

Based on the practical aspects summarized in Table 1, MSPDE demonstrates moderate simplicity as the procedure is conceptually straightforward but requires careful manual blending and packing steps.^78^ Its rapidness is moderate since extraction and cleanup occur simultaneously, yet optimization and solvent elution steps may extend processing time. The technique exhibits moderate selectivity, largely governed by the choice of dispersant and sorbent materials rather than highly specific interactions.^77^ In terms of versatility, MSPDE is moderate, performing particularly well for solid and semisolid matrices but less consistently in highly aqueous systems. Regarding sustainability, MSPDE scores moderate in greenness as it uses organic solvents, though typically in lower volumes compared to traditional exhaustive extraction techniques.^78^ Overall, MSPDE remains a practical and adaptable option for pharmaceutical extraction from complex solid environmental matrices, particularly when integrated extraction and cleanup are desirable in a single step.

#### 3.2.5 Stir-Bar Sorptive Extraction

SBSE is a miniaturized, largely solvent-free sample preparation method for the extraction and concentration of organic compounds from aqueous samples, in which solutes are extracted into a polydimethylsiloxane polymer coating (PDMS) on a stir bar (Figure 6c).^159,160^ The technique has been recognized as an emerging online sample preparation method that can overcome the weaknesses and shortcomings of traditional sample preparation methods.^148^ As such, some researchers have been using SBSE in extracting numerous pharmaceuticals from environmental matrices (Table 2).

Klančar et al.^140^ have successfully extracted a wide range of pharmaceuticals, which include amitriptyline, azithromycin, desipramine, diazepam, donepezil, escitalopram, fluoxetine, haloperidol, imatinib, clomipramine, loperamide, loratadine, metoprolol, promethazine, propranolol, raloxifene, risperidone, selegiline, sertraline, tramadol, triclosan, venlafaxine, verapamil, ziprasidone, bromazepam, carbamazepine, and clonazepam, from wastewater samples using the SBSE approach, with reported recoveries ranging from 25.00 to 85.0%. The wider range of the reported recoveries is mainly attributed to the physicochemical property differences in the targeted pharmaceuticals. The least recovered pharmaceutical, propranolol (19.4% extraction efficiency), is relatively polar, while the most recovered, haloperidol (100.6% extraction efficiency), is less polar and more hydrophobic. Carbamazepine, β estradiol, 3-(4-methylbenzylidene) camphor, benzophenone-1, and ibuprofen have been recovered from spiked water samples using SBSE at 71.4–83.2% recoveries.^132^

Researchers have also noted that SBSE can enrich volatile and semi-volatile organic compounds with high extraction efficiency compared to SPME but requires longer extraction time.^149^ This has allowed the technique to be applied effectively in environmental analysis, mainly with gas chromatography–mass spectrometry (GC–MS).^149^ Generally, SBSE shows limited recoveries for polar solutes.^160^ Although several more polar coatings for SBSE were developed, these extraction phases are mostly not compatible with thermal desorption and have inferior performance characteristics related to robustness, bleeding, and stability compared to PDMS.^160^

From a practical perspective (Table 1), SBSE is relatively simple, with a straightforward operational procedure, but requires specialized stir bars and thermal desorption or solvent back-extraction systems.^75^ Its rapidness is moderate since equilibrium may require longer stirring times compared to other microextraction techniques. SBSE exhibits high selectivity, particularly for non-polar and semi-volatile compounds that strongly partition into the polymer coating.^76^ The versatility is moderate as it performs well with hydrophobic analytes but less effectively with highly polar pharmaceuticals. In terms of sustainability, SBSE scores high in greenness, is largely solvent-free, and generates minimal chemical waste. Furthermore, reusability is high as stir bars can be reused multiple times after proper cleaning and conditioning without a significant loss of performance.^75^

Overall, SBSE is particularly powerful for the extraction of volatile and semi-volatile organic compounds, especially hydrophobic pharmaceuticals. Its greater sorptive capacity provides superior enrichment compared to fiber-based techniques, making it highly suitable for trace-level analysis of volatile compounds in environmental samples, particularly when coupled with GC–MS.

#### 3.2.6 Emerging Solid-Phase Extraction-Based Techniques

More developments are still emerging in the SPE category of the sample preparation. Among the emerging solid-phase-based miniaturized extraction techniques are microextraction by packed sorbent (MEPS), polymer monolith microextraction (PMME), solid-phase dynamic extraction (SPDE) (Figure 6d), and fabric phase sorptive extraction (FPSE). MEPS represents a kind of miniaturization of conventional SPE from milliliters to microliters bed volumes.^148^ It has been developed as one of the novel extraction techniques resulting from miniaturization and automation.^125,161^ PMME^162^ and SPDE^163^ techniques have also been utilized in recent pharmaceutical extraction studies as miniaturized variants of conventional SPE techniques. PMME has been successfully implemented in extracting pharmaceuticals from different matrices, including water, food, and urine.^79,80^ The highly porous structure of polymers used in PMME enhances interaction with analytes, thereby increasing the extraction capacity.^79^ FPSE is a sorbent-based sorptive microextraction technique that utilizes organic polymers immobilized on fabric substrates.^164^ FPSE is among the emerging miniaturized and environmentally friendly techniques rapidly replacing classical sample preparation methods.^164,165^

### 3.3 Emerging Hybrid Extraction Techniques

Emerging hybrid extraction techniques integrate complementary extraction principles to overcome the limitations associated with individual methods. In general, conventional or sorbent-based extraction is first employed to isolate or clean up the analytes, followed by a miniaturized extraction step to improve enrichment and analytical sensitivity.^167^ Recent developments increasingly incorporate magnetic sorbents, ionic liquids, deep eutectic solvents, and other green extraction materials to enhance selectivity while reducing solvent consumption.^168^ Figure 7 shows a schematic representation of an emerging hybrid extraction technique (SPE-DLLME). The schematic illustrates a workflow of a typical hybrid technique for pharmaceutical analysis in environmental samples. The sample is first pre-treated and passed through an SPE cartridge, where the target pharmaceuticals are retained while most matrix interferences are removed. The analytes are then eluted using a small volume of organic solvent and subjected to DLLME, in which an extraction solvent and disperser solvent form a fine cloudy dispersion that rapidly concentrates the analytes. Following centrifugation, the enriched extract is collected and analyzed by chromatographic techniques such as HPLC or LC–MS. By combining the cleanup capability of SPE with the rapid enrichment of DLLME, the hybrid approach improves extraction efficiency, reduces matrix effects, and enhances analytical sensitivity for trace pharmaceutical determination.

**Figure 7:**
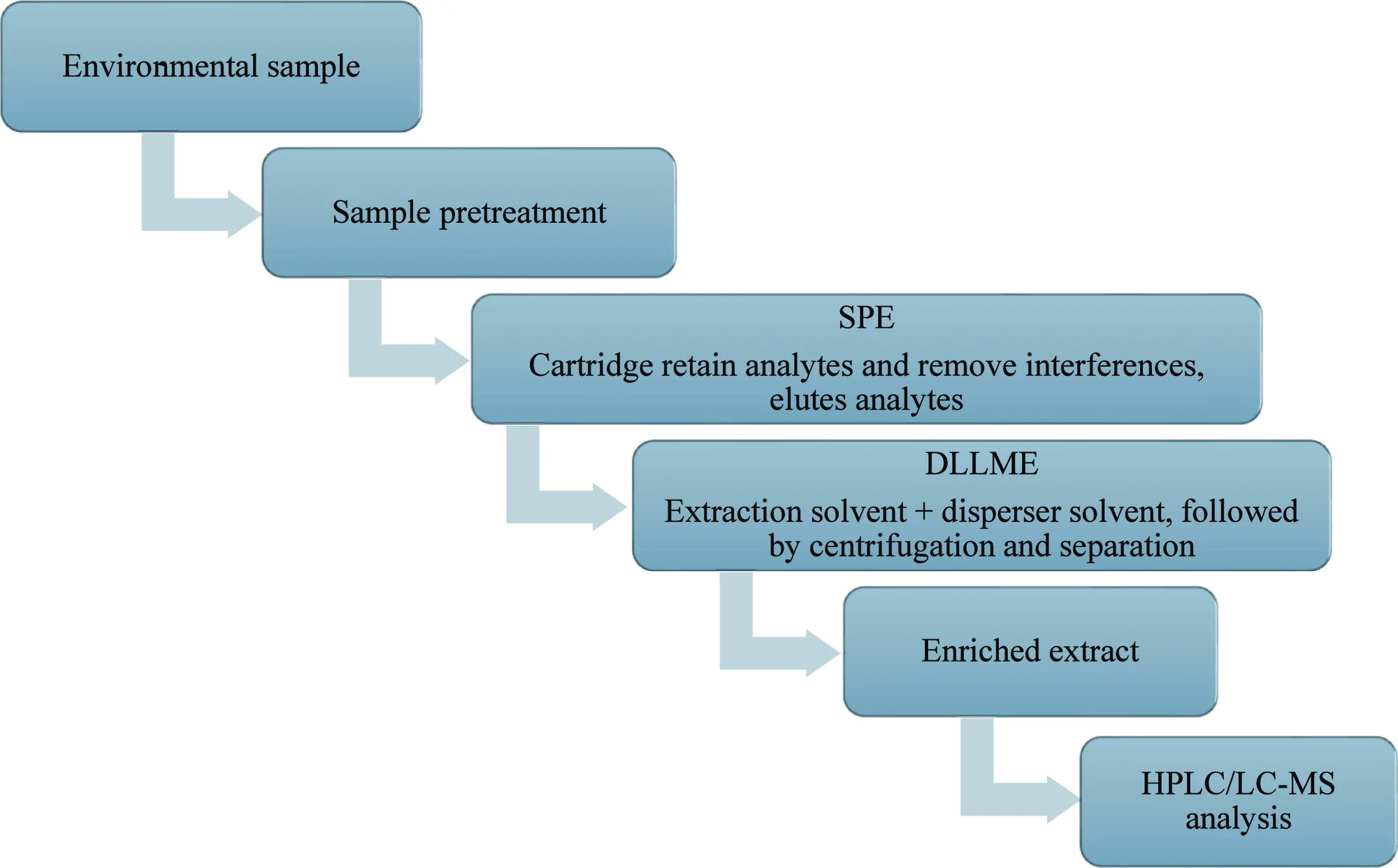
Schematic representation of a hybrid extraction technique: SPE-DLLME.

Although the schematic (Figure 7) illustrates the SPE-DLLME hybrid approach, the other hybrid extraction techniques follow the same general principle. The specific workflow depends on the extraction techniques being integrated and the sequence in which they are coupled. In most cases, one technique is initially employed to isolate, clean up, or preconcentrate target analytes, after which a second complementary technique is applied to further enhance selectivity, enrichment, or analytical sensitivity.^169^ Most of these hybrid approaches have been applied in extraction and preconcentration of other contaminants, including polycyclic aromatic hydrocarbons,^46^ pesticides,^45^ and dyes,^48^ and are still being investigated on pharmaceuticals, with trials conducted on different matrices including industrial wastewater, food, and surface water.^43,170^

Recent developments have also seen the emergence of minimal sample pretreatment hybrid analytical techniques that integrate simplified sample handling with ambient- or direct-ionization mass spectrometry. Examples include indirect solution analysis coupled with desorption electrospray ionization (ISA-DESI) and laser desorption ionization (ISA-LDI), which have recently been demonstrated for the rapid determination of persistent contaminants such as per- and polyfluoroalkyl substances (PFASs) with little or no conventional sample preparation.^171^ Similar advances include paper spray ionization mass spectrometry (PSI-MS),^172,173^ coated blade spray mass spectrometry (CBS-MS),^174^ and solid-phase microextraction coupled with direct analysis in real-time mass spectrometry (SPME-MS),^175,176^ all of which combine extraction, enrichment, and ionization into streamlined workflows. Although these hybrid approaches have been predominantly explored for analytes like lipid profiles, proteins, and PFASs in forensic,^173^ clinical,^172,174^ and food^176^ samples, their rapid analysis, reduced solvent consumption, and minimal sample handling make them promising candidates for the determination of pharmaceuticals in complex environmental matrices. Table 4 summarizes some examples of emerging hybrid extraction techniques that can be used for pharmaceutical analysis, including their integration principles and key practical features.

**Table 4:** Emerging Hybrid Extraction Techniques Applicable for Pharmaceutical Analysis^a^

Hybrid extraction technique	Extraction phases combined	Main integration principle	Major advantages	Typical limitation	Reference
LLE-SPE	Liquid + Solid	LLE for bulk extraction followed by SPE cleanup and concentration	Improved cleanup, higher enrichment, reduced matrix effects	More preparation steps	^170^
SPE-LLME	Solid + Liquid	SPE preconcentration followed by LLME for further enrichment	Excellent sensitivity, reduced solvent consumption	Requires optimization of both stages	^43^
SPE-DLLME	Solid + Liquid	SPE cleanup followed by rapid DLLME	High recoveries and enrichment factors	Increased procedural complexity	^44^
MSPE-DLLME	Solid + Liquid	Magnetic sorbent extraction followed by DLLME	Fast operation, cleaner extracts	Magnetic sorbent preparation may be required	^45^
DLLME-SPE	Liquid + Solid	DLLME preconcentration followed by SPE purification	Improved extract purity	Additional processing step	^46^
SPE–HS–SPME	Solid + Solid	SPE enrichment followed by headspace SPME	Excellent for volatile and semi-volatile compounds	Limited applicability to non-volatile analytes	^47^
LLME-MSPE	Liquid + Solid	LLME followed by magnetic sorbent cleanup	Reduced matrix interferences	Requires compatible magnetic sorbents	^48^
MSPE-SBSE	Solid + Solid	Magnetic extraction followed by stir-bar sorptive extraction	Improved selectivity and reusability	Longer extraction time	^49^
IL-DLLME-MSPE	Liquid + Solid	Ionic liquid-based DLLME followed by magnetic SPE	Greener extraction, high selectivity, low solvent consumption	Cost and availability of ionic liquids	^50^
MSPDE-DLLME	Solid + Liquid	MSPDE sample disruption followed by DLLME enrichment	Suitable for difficult matrices	Multi-step optimization required	^51^

a Abbreviations: LLE = liquid–liquid extraction; SPE = solid-phase extraction; LLME = liquid–liquid microextraction; DLLME = dispersive liquid–liquid microextraction; MSPE = magnetic solid-phase extraction; HS-SPME = headspace solid-phase microextraction; SBSE = stir-bar sorptive extraction; IL = ionic liquid; MSPDE = matrix solid-phase dispersion extraction.

## 4 Critical Insights

Several critical aspects regarding conventional and modern miniaturized extraction and preconcentration techniques can be drawn from this review. It is important to note that no single extraction or preconcentration technique can be considered to be universally superior for environmental sample analysis. The performance of both conventional and miniaturized techniques is highly dependent on the physicochemical properties of the target analytes, such as polarity, pKa, molecular weight, and hydrophobicity as well as the complexity of the sample matrix. This is clearly demonstrated by the wide variability in reported recoveries and enrichment factors for different pharmaceuticals extracted by using the same technique. For instance, conventional LLE and SPE showed recovery ranges of 9–120%, while miniaturized techniques showed recovery ranges of 9.4–229.0%.

Another important observation is the influence of sample matrix complexity on the extraction performance. Relatively clean matrices, such as surface water and groundwater, generally allow higher and more consistent recoveries because they contain fewer interfering substances. In contrast, complex matrices including wastewater, sewage sludge, sediments, and landfill leachates contain dissolved organic matter, suspended solids, salts, and co-existing contaminants that can compete with target pharmaceuticals during extraction or contribute to matrix effects. Consequently, these matrices often require more selective extraction techniques and careful optimization of extraction conditions to achieve a reliable analytical performance. Although miniaturized techniques have demonstrated excellent recoveries and enrichment factors in real environmental applications, their robustness largely depends on appropriate optimization of parameters such as pH, extraction time, solvent or sorbent selection, and sample cleanup. Therefore, extraction method selection should consider not only the analytical performance but also the complexity of the target environmental matrix and the intended application.

Conventional LLE and SPE showed wider applicability, mostly because of the possibility of switching from one solvent to another. They are generally compatible with a wide array of solvents and sorbents, enabling the extraction of pharmaceuticals with a variety of physicochemical properties. These techniques remain relatively accessible and operationally familiar in most of the analytical laboratories.

Miniaturized techniques generally offer improved enrichment factors, reduced solvent consumption, and faster extraction times but are not without limitations. Techniques such as SDME, HF-LPME, DLLME, SPME, MSPE, and SBSE exhibit analyte-specific selectivity and often require the careful optimization of extraction parameters. Some miniaturized methods may still depend on organic solvents or experience matrix effects in complex samples, such as wastewater.

Beyond recovery and enrichment trends, a comparative performance assessment presented as a heatmap in Figure 8 provides further insight by ranking the techniques based on score-ranking of practical aspects: simplicity, rapidness, selectivity, versatility, greenness, and reusability, derived from reported previous studies.^60–80^ The comparison shows that from the reviewed papers, conventional LLE scores low in greenness and reusability but moderate in versatility. SPE demonstrates high selectivity and versatility but moderate rapidness and greenness. Among the reviewed miniaturized techniques, SDME and DLLME stand out for high rapidness, while HF-LPME and MSPE demonstrate high selectivity. SBSE exhibits strong reusability and greenness due to its solventless nature and reusable stir bars. MSPE combines relatively high selectivity with operational convenience due to magnetic separation.

**Figure 8:**
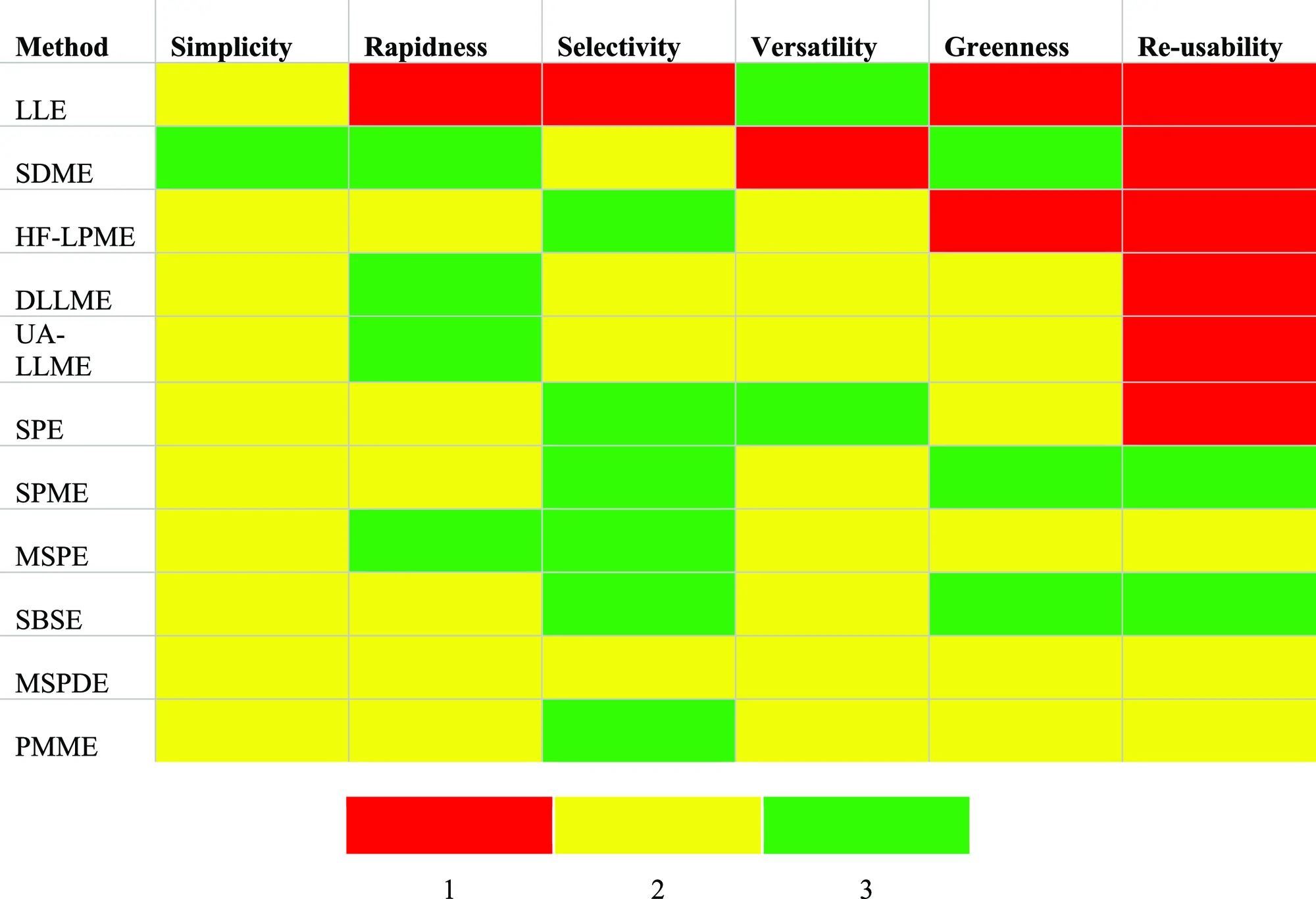
Literature-informed qualitative comparison of the practical performance of pharmaceutical extraction techniques. Numerical scores (1–3) were assigned from qualitative descriptors reported in the cited literature and are intended only for comparative visualization rather than quantitative ranking or statistical analysis. Scoring interpretation: simplicity: 1 = complex setup, multiple steps, 2 = moderate operational steps, 3 = easy setup, minimal handling; rapidness: 1 = time-consuming, 2 = moderate extraction time, 3 = fast extraction and processing; selectivity: 1 = poor discrimination; matrix interference likely, 2 = moderate selectivity, 3 = high specificity toward target analytes; versatility: 1 = limited analyte range, 2 = moderate applicability, 3 = broad analyte and matrix compatibility; greenness: 1 = high solvent use, hazardous solvents, 2 = reduced solvent use, 3 = minimal solvent; environmentally friendly; reusability: 1 = single use, 2 = limited reuse, 3 = multiple reuses without major performance loss.

Across the reviewed studies, several consistent trends emerged, irrespective of the target pharmaceutical. Conventional techniques generally handle larger sample volumes and offer broader applicability across different pharmaceutical classes, whereas miniaturized approaches consistently reduce solvent consumption and frequently achieve higher enrichment factors. Wastewater and sludge matrices generally require more selective extraction procedures than relatively clean matrices such as surface water or groundwater because of increased matrix interferences. Similarly, the extraction performance was influenced by analyte polarity and hydrophobicity, with no single technique consistently outperforming others across all pharmaceutical classes. These observations reinforce that extraction method selection should be guided by the combined influence of analyte characteristics, sample matrix, analytical objectives, and practical considerations rather than by recovery alone.

From a practical standpoint, researchers prioritizing speed and minimal solvent consumption may prefer SDME or DLLME. Where high selectivity and cleaner extracts are critical, SPE, MSPE, or HF-LPME may be more suitable. If reusability and environmental sustainability are major considerations, SBSE and MSPE offer advantages. For applications requiring broad analyte coverage, conventional SPE and LLE remain viable options, despite their solvent demands.

It is also crucial to note that continuous methodological developments have significantly improved the extraction efficiency, selectivity, and environmental sustainability. The incorporation of ionic liquids, deep eutectic solvents, magnetic sorbents, and hybrid extraction approaches reflects a clear trend toward greener and more efficient sample preparation. These developments are likely to further refine the performance trade-offs among simplicity, selectivity, speed, and sustainability. Looking ahead, further progress is expected to be driven by the development of more selective and robust sorbent materials, greener extraction solvents, and improved hybrid extraction strategies capable of addressing increasingly complex environmental matrixes. Advances in miniaturized and automated sample preparation, coupled with portable analytical platforms, are also likely to facilitate rapid on-site monitoring while reducing the analysis time and solvent consumption. As pharmaceutical pollution continues to diversify, future extraction methods will need to accommodate a broader range of emerging contaminants, transformation products, and multiresidue analyses without compromising analytical performance. From a practical perspective, wider routine adoption of these techniques will depend not only on analytical performance but also on method standardization, reproducibility across laboratories, cost effectiveness, and compatibility with existing analytical instrumentation. Therefore, researchers must remain informed about emerging techniques and evolving trends in order to select the most suitable method that aligns with their analytical goals and performance priorities.

## 5 Recommendations

Based on the findings of this review, researchers are advised to adopt a case-by-case approach when selecting extraction and preconcentration techniques for pharmaceutical analysis in environmental samples. Since no technique can be classified as the “best,” the choice of the extraction method should be guided primarily by the properties of the target pharmaceutical(s), including polarity, and ionization behavior since these directly affect recovery and enrichment. The nature of the sample matrix is also an important factor as complex matrices such as wastewater and sludge often require techniques with higher selectivity and effective cleanup capability. More importantly, the expected environmental concentration levels should be taken into consideration. Researchers should also consider the practical performance aspects of the extraction method, including simplicity, rapidness, selectivity, versatility, greenness, and reusability. These operational criteria can significantly influence the suitability of the method for executing experimental work.

It is also important to clearly define the goal of the extraction procedure before the selection of the method. For studies requiring high enrichment factors and trace-level detection, miniaturized techniques such as HF-LPME, DLLME, MSPE, or SBSE may be more appropriate. If environmental sustainability is a priority, then greener options such as SPME and SBSE may be preferable. Conversely, when robustness, method simplicity, and wider analyte application are prioritized, conventional SPE or LLE may still be suitable, particularly for routine monitoring.

Researchers are further encouraged to evaluate extraction methods holistically by considering not only recovery and enrichment factors but also practical aspects such as matrix complexity, sample volume requirements, solvent consumption, environmental impact, cost, sorbent availability, compatibility with analytical instrumentation, and overall operational practicality. Techniques with high reusability (e.g., SBSE and SPME) may reduce long-term operational costs, whereas methods with low reusability (e.g., LLE and DLLME) may increase consumable expenses despite their analytical strengths. Optimization of experimental parameters should not be overlooked as extraction efficiency and recovery are highly sensitive to conditions such as the pH, extraction time, and solvent composition. Finally, staying up-to-date with new developments is encouraged. Emerging developments, including greener extraction materials and solvents as well as hybrid extraction approaches, are important for improving analytical performance and making pharmaceutical environmental analysis more sustainable. Ultimately, balancing analytical performance with practical criteria such as simplicity, speed, environmental impact, and material reusability will lead to more informed and sustainable method selection decisions.

## 6 Limitations of the Review

Although this review provides a comprehensive comparison of conventional and miniaturized extraction techniques for pharmaceuticals in environmental samples, some limitations should be acknowledged. First, the review was intentionally limited to representative peer-reviewed studies reporting quantitative extraction performance for pharmaceuticals in environmental matrixes. Consequently, the findings should not be interpreted as a systematic synthesis of all of the published studies. Furthermore, direct comparisons between studies are inherently challenging because reported recoveries and enrichment factors are influenced by differences in pharmaceutical classes, environmental matrices, extraction conditions, sorbent or solvent selection, sample volumes, analytical instrumentation, and other experimental variables. Accordingly, the comparisons presented in this review should be regarded as representative trends interpreted within the context of studies that had already established the analytical reliability of their respective methods.

Another limitation is that recovery was more consistently reported than the enrichment factor across the reviewed studies. Consequently, much of the comparative discussion places greater emphasis on recovery, which is also the most widely reported and commonly accepted indicator of extraction performance in analytical chemistry, while enrichment factors were discussed where sufficient data were available.

In addition, the practical performance assessment based on simplicity, rapidness, selectivity, versatility, greenness, and reusability was developed from descriptive information reported in the reviewed literature. The qualitative scores assigned to each technique therefore represent a literature-informed synthesis intended to summarize general practical characteristics rather than a statistical ranking or a formal meta-analysis. Despite these limitations, this review provides a balanced and application-oriented comparison that integrates analytical performance with practical considerations to support informed method selection for pharmaceutical monitoring in environmental systems.

## 7 Conclusions

This review has critically evaluated the performance of conventional and miniaturized extraction and preconcentration techniques for pharmaceutical analysis in environmental samples. Conventional methods, particularly LLE and SPE, remain valuable due to their robustness and wide applicability, but they are increasingly challenged by issues related to solvent consumption, time requirements, and limited selectivity. Miniaturized techniques such as SDME, HF-LPME, DLLME, SPME, MSPE, and SBSE have demonstrated significant advantages, including higher enrichment factors, reduced solvent usage, and improved sensitivity, making them attractive alternatives for trace-level pharmaceutical determinations. However, the review clearly shows that extraction efficiency and recovery vary widely depending on the analyte and matrix, emphasizing that no single technique is universally optimal. Reliable performance in real environmental applications ultimately depends on selecting and optimizing extraction methods according to matrix complexity, in addition to analyte properties and analytical objectives. Beyond recovery and enrichment trends, the practical comparative performance of the extraction methods further highlights that each technique has distinct strengths and limitations in terms of simplicity, rapidity, selectivity, versatility, greenness, and reusability. These practical considerations are equally important for laboratory implementation and long-term sustainability. Ongoing advancements in extraction materials, green solvents, and hybrid approaches continue to enhance the analytical performance. Importantly, modern method selection should not only aim for high recovery or enrichment but also balance operational simplicity, environmental impact, cost efficiency, and material reusability. Reliable and effective method selection in environmental pharmaceutical analysis should therefore be guided by project objectives, sample characteristics, available instrumentation, and the broader practical performance profiles of each technique.
